# Supplementary figures and images for: DEXOM: Diversity-based enumeration of optimal context-specific metabolic networks
Source: PLoS Comput Biol. 2021 Feb 11;17(2):e1008730. doi: 10.1371/journal.pcbi.1008730 (PMC7904180; doi:10.1371/journal.pcbi.1008730)

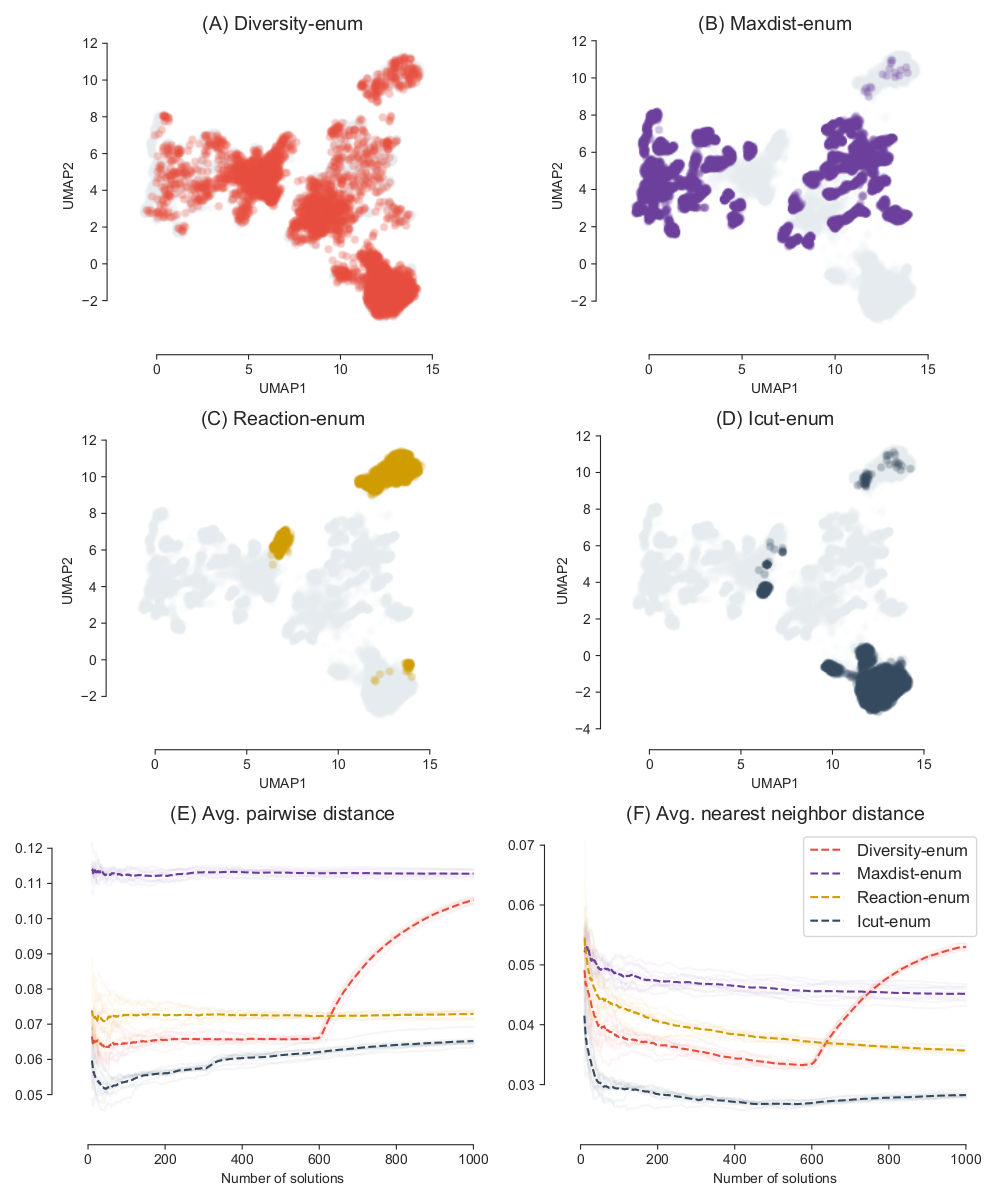

Supplement: S1 Fig — (TIF) [file pcbi.1008730.s001.tif]

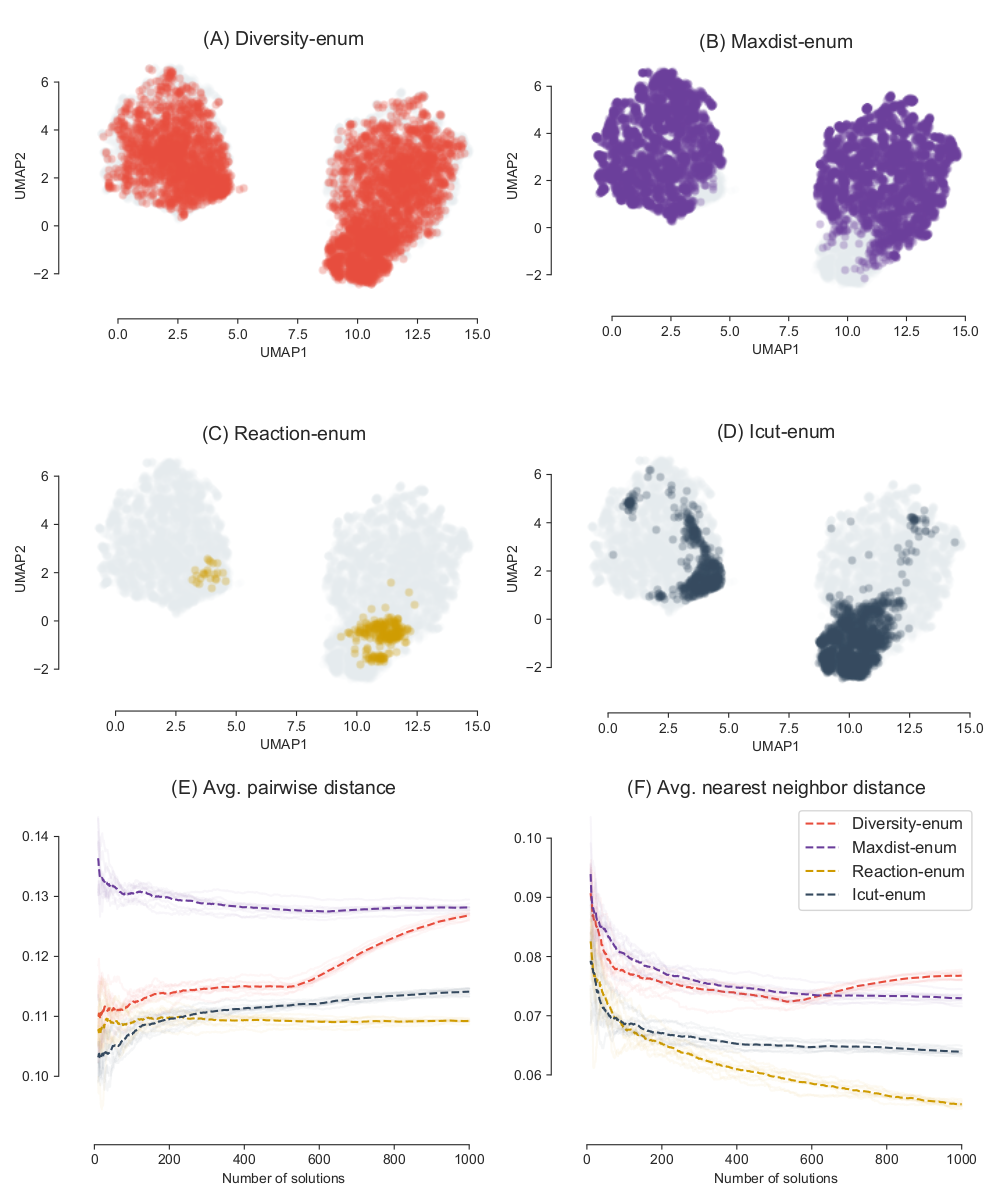

Supplement: S2 Fig — (TIF) [file pcbi.1008730.s002.tif]

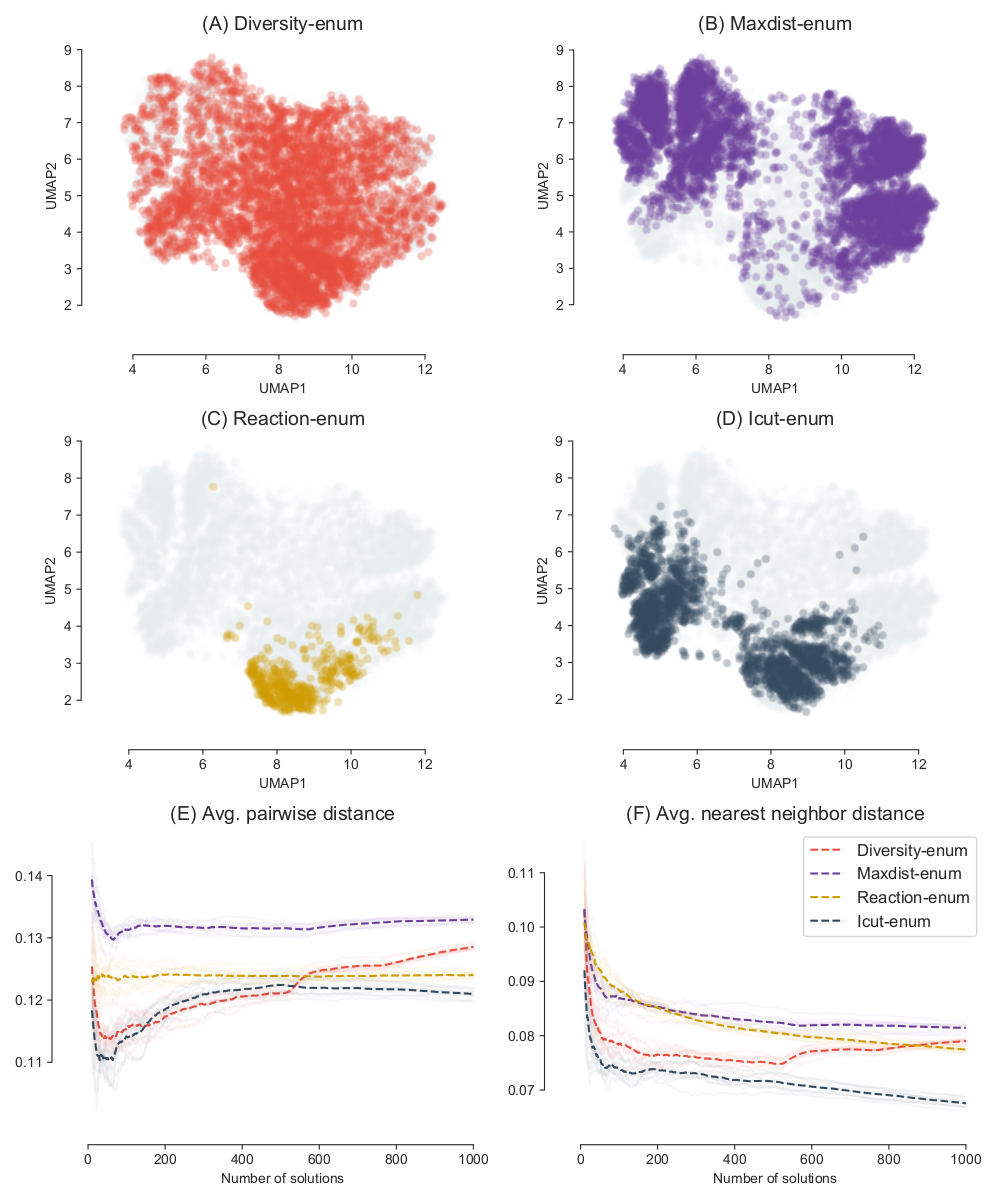

Supplement: S3 Fig — (TIF) [file pcbi.1008730.s003.tif]

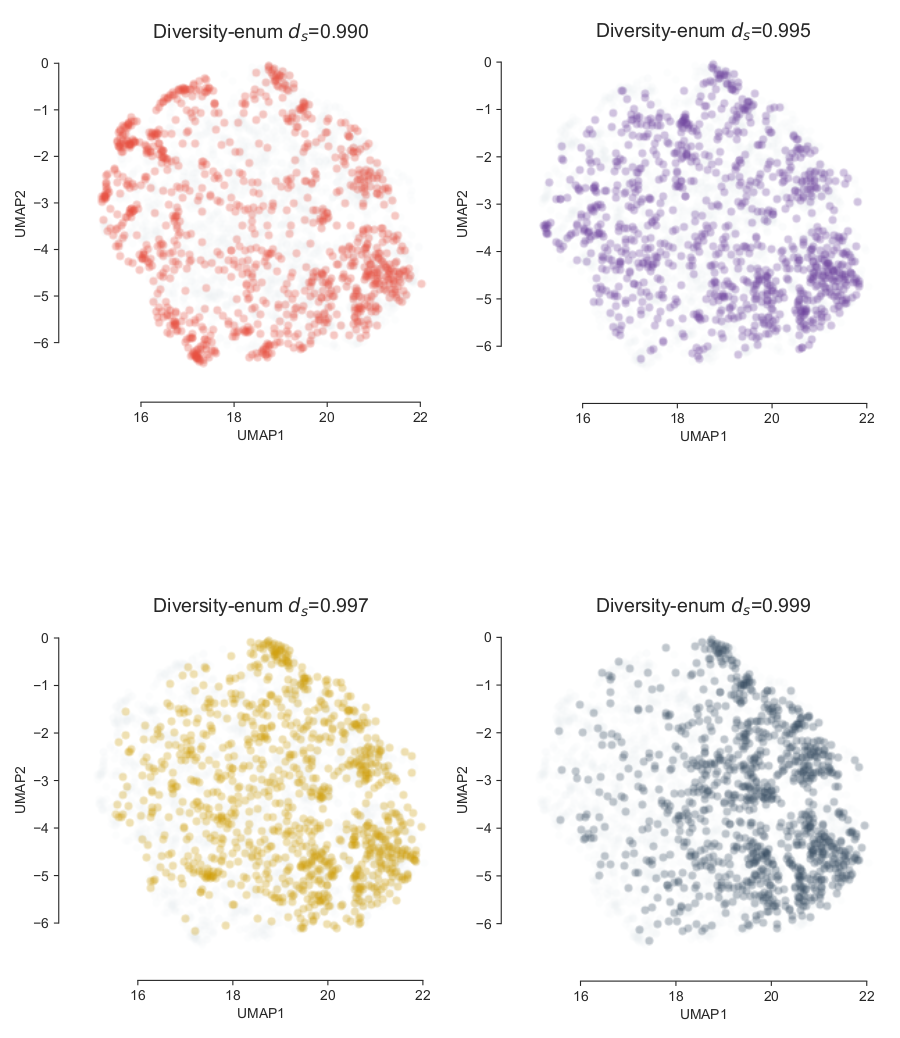

Supplement: S4 Fig — Values closer to 1 make the enumeration progress more slowly from closer to distant optimal solutions, discovering more proximate and intermediate solutions. When the value is lower (e.g. 0.990), the enumeration reaches the distant solutions more quickly, enumerating more solutions at the extremes. (TIF) [file pcbi.1008730.s004.tif]

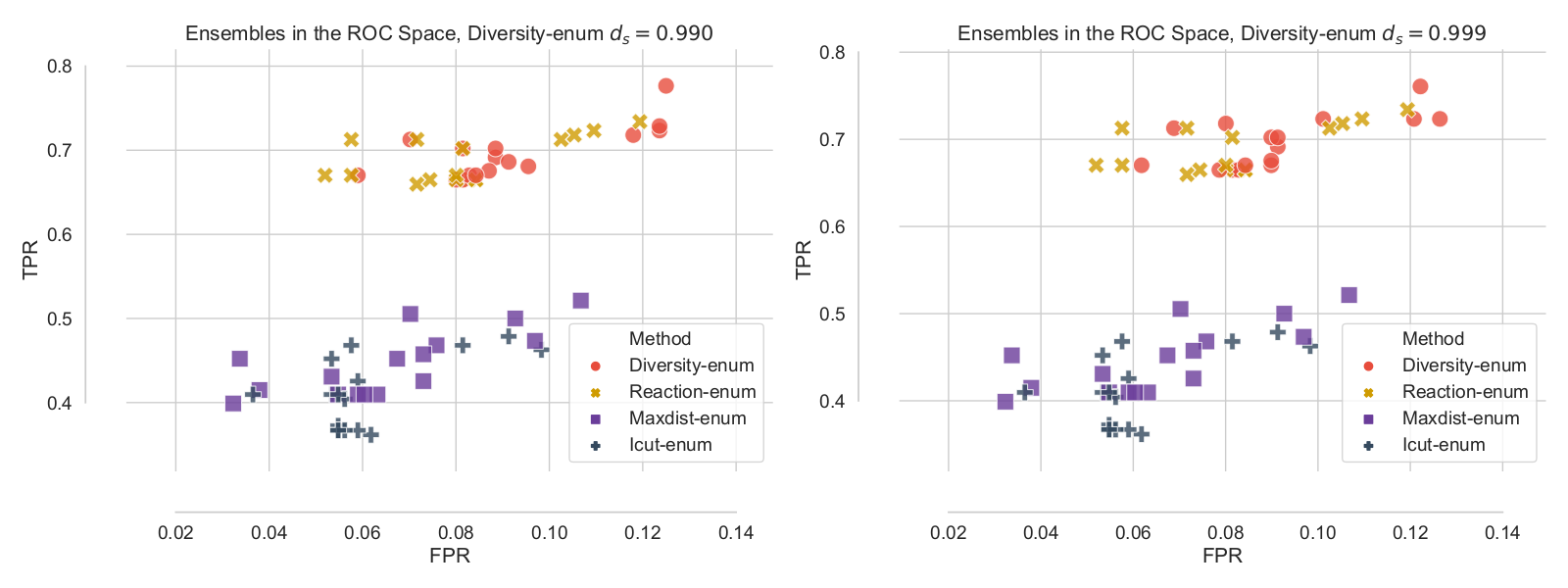

Supplement: S5 Fig — Analysis was repeated with parameter values ds = 0.990 and ds = 0.999 instead of the default value (ds = 0.995). The results show almost no variation in terms of the TPR and FPR of the ensembles. (TIF) [file pcbi.1008730.s005.tif]

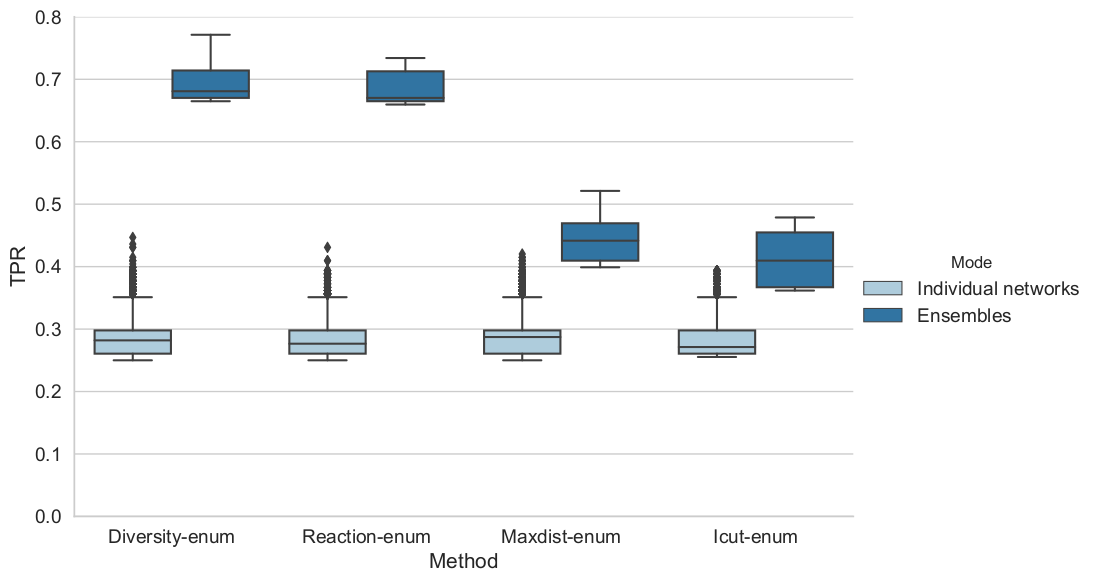

Supplement: S6 Fig — Results show 1) the variability in the predictions of true essential genes by individual networks, and 2) the result of the ensemble for each method. Although variation of results of the individual solutions enumerated with each method are similar, results of the ensemble greatly differ between methods. This indicates that although the individual networks predict a similar number of true positive essential genes, these sets present less overlapping in networks enumerated with Diversity-enum and Reaction-enum, and therefore the overall TPR of the ensembles generated by these methods is better. (TIF) [file pcbi.1008730.s006.tif]

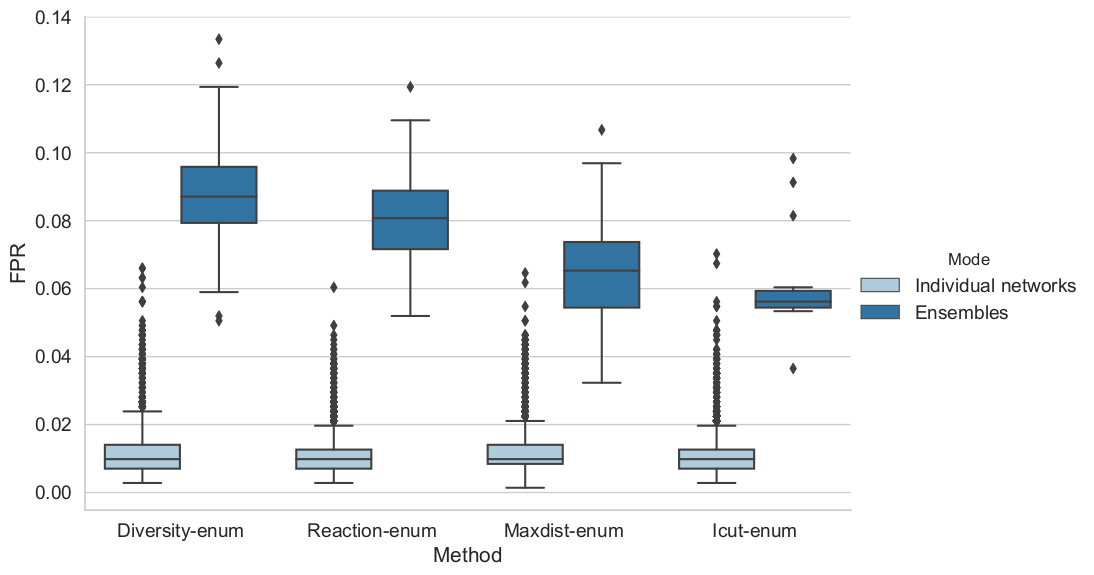

Supplement: S7 Fig — Results show, as for the TPR, an increase of the FPR of the ensembles, more pronounced for Diversity-enum and Reaction-enum, since there is always a trade-off between both measurements: increasing the predictions of true positives comes with a higher rate of false positives. (TIF) [file pcbi.1008730.s007.tif]
