# Supplementary material for: DEXOM: Diversity-based enumeration of optimal context-specific metabolic networks
Source: PLoS Comput Biol. 2021 Feb 11;17(2):e1008730. doi: 10.1371/journal.pcbi.1008730 (PMC7904180; doi:10.1371/journal.pcbi.1008730)
Supplement: S1 Appendix — PDF including: 1) computational time of the different enumeration techniques; 2) distribution of predicted essential genes among methods; and 3) variability in the detection of enriched pathways between the different methods. (PDF) [file pcbi.1008730.s009.pdf]

Supporting Information

DEXOM: Diversity-based enumeration of optimal context-specific metabolic networks

Pablo Rodríguez-Mier[1], Nathalie Poupin[1], Carlo de Blasio[2,3], Laurent Le Cam[2,3], Fabien Jourdan[1*]

1 Toxalim (Research Centre in Food Toxicology), Université de Toulouse, INRAE, ENVT, INP-Purpan, UPS, Toulouse, France.
2 IRCM, Institut de Recherche en Cancérologie de Montpellier, INSERM U1194, Université de Montpellier, Institut régional du Cancer de Montpellier, Montpellier, France.
3 Equipe Labellisée par la Ligue contre le Cancer, Paris, France.

* fabien.jourdan@inrae.fr

Computation time of the different enumeration methods

One factor that affects the performance is the number of variables of the MILP problem, and this also depends on the threshold selected. For example, if the number of genes is 1,000 and the threshold for the lowly expressed genes and highly expressed genes is [0.10, 0.90], then only 20% of the genes (200 genes) are used, whereas if the threshold is [0.25, 0.75], 50% of the genes are used. Mapping a bigger set of genes into the networks will translate into larger sets of R_H reactions to maximize and R_L reactions to minimize, and thus bigger MILP problems with more binary variables to optimize.

Fig. 1 shows the number of solutions over time for the thresholds used in the evaluation that take the minimum number of genes ([0.10, 0.90], 20% of genes) and the maximum number of genes ([0.25, 0.75], 50% of genes). Fig. 1A shows the solutions obtained over time for the case when only 20% of the genes is classified as highly expressed or lowly expressed. In this situation, `Diversity-enum`, `Maxdist-enum` and `Icut-enum` follow a similar trend. `Reaction-enum` is the fastest method, taking less than 10 minutes to finish the enumeration. This is due to the fact that solving each MILP problem has almost no extra cost with respect to the original problem, since only a single constraint to force the inclusion or removal of one reactions is added to the original problem. In contrast, the other methods solve a more complex optimization problem, and the number of constraints grows monotonically, making more difficult the enumeration over time.

Differences between the techniques become more extreme as the number of genes increases. Fig. 1B shows again the solutions obtained over time but for the threshold [0.25, 0.75], in which 50% of genes are mapped in the networks. `Reaction-enum` is again the fastest method, followed by `Icut-enum`, `Diversity-enum` and `Maxdist-enum`. Concretely, `Reaction-enum` takes again

less than 10 minutes to complete, whereas `Icut-enum` takes around 1.5 hours, `Diversity-enum` 5 hours and `Maxdist-enum` around 8 hours. `Maxdist-enum` is heavily penalized by the increase in the number of selected genes. This is due to the fact that `Maxdist-enum` searches for the most distant vector at each step, and the dimension of this vector correspond to the number of reactions in the R_H and R_L sets, which are bigger in this case. `Diversity-enum` is less penalized since at the beginning of the search, only a few components of the vector are used to maximize the distance. However, the performance degrades as the distance increases over time, until the distance is maximal. At this point, the performance of `Diversity-enum` is similar to `Maxdist-enum`. The total time for the evaluation, including all methods and calculation of in-silico predictions of essential gene for each optimal network took around 150 hours in an Intel Core i7 4790 @ 3.60 GHz (8 threads) with CPLEX 12.8 and Matlab 2015 academic.

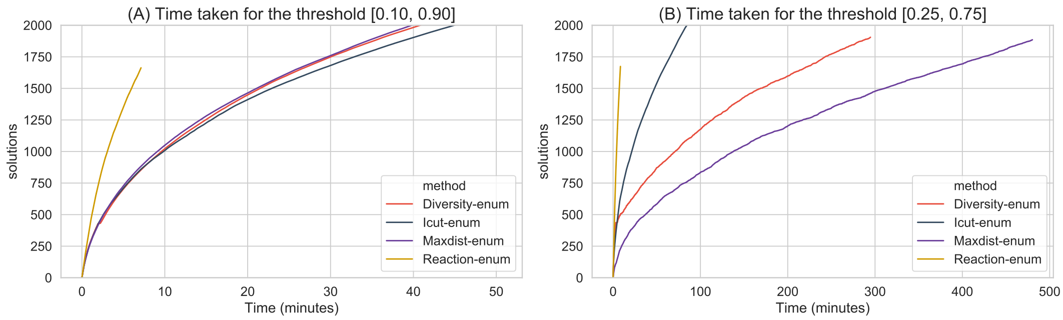

Figure 1: Total number of solutions enumerated over time for the different techniques for two different thresholds. A) Using a gene threshold of [0.10, 0.90]. B) Using a gene threshold of [0.25, 0.75].

Distribution of predicted essential genes among methods

Figure 2 shows the intersections of the correctly predicted essential genes between the different methods. In total, 95 essential genes are commonly predicted by the four methods, from which 47 are always classified as essential by all the networks (genes that are always essential in the unconstrained full Yeast 6 model), and the remaining 48 genes are classified as essential in different proportions depending on the method. The second largest intersection corresponds to the intersection of the `Diversity-enum` and `Reaction-enum`, sharing in total 36 true positives (none of them detected by the other methods). This might indicate that a big fraction (around 19%) of the context-specific essential genes (aerobic genes not essential in the full Yeast 6 GSMN) can be detected by alternative context-specific networks that are not too different on average (the alternative networks generated by perturbing single reactions). Also, `Diversity-enum` detects 3 essential (auxotrophic) genes (YGL055W, YGR155W, YAL012W) that are also detected only by `Maxdist-enum` but not by `Reaction-enum` or `Icut-enum`, highlighting also similarities between the way in which `Maxdist-enum` and `Diversity-enum` explore solutions. Interestingly, `Icut-enum`, although it is the technique that generates more similar solutions and therefore detects fewer different essential

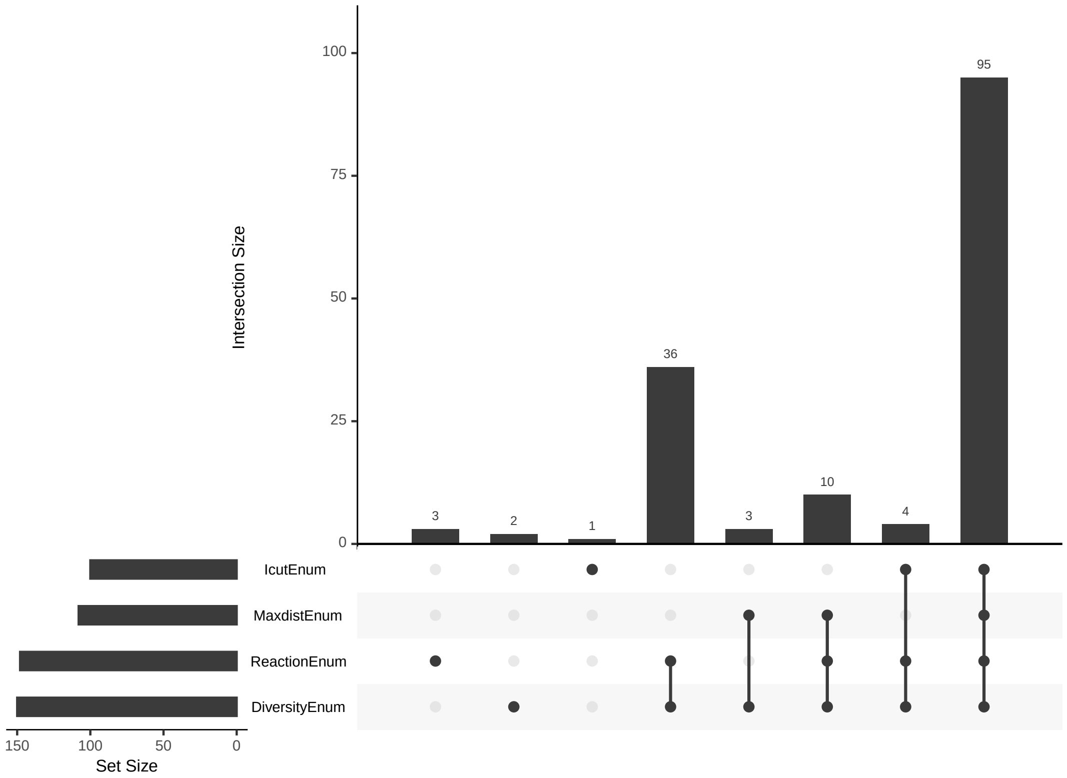

Figure 2: Intersection of the correctly predicted essential genes (true positives) using the Yeast 6 model [1] and the UpSetR package [2]. The first three vertical bars show the number of unique essential genes detected by the `Reaction-enum` method (3 genes), `Diversity-enum` method (2 genes), and `Icut-enum` method (1 gene). The remaining vertical bars show the intersections between two, three and the four methods.

genes, it correctly predicts one essential gene (YHR128W) that was not predicted by any of the alternative networks generated with the other techniques.

Figure 3 shows the top 50 most correctly predicted genes, after removing the 47 genes that are always correctly classified by all the networks/methods. The Y-axis shows the average proportion of times that each gene was correctly predicted as essential, and the error bars show the variability between methods (minimum and maximum value detected). As can be seen, some genes are more likely to be classified as essential. This could be explained by the fact that given the distribution of genes and the topology of the network itself, the elementary flux modes of the possible solutions contain with a higher frequency the reactions associated to those genes. However, there might be other factors that can affect these proportions. For example, given that the methods do not uniformly sample the space of possible solutions, samples generated by these methods might be also biased towards some particular type of solutions. It is thus important to emphasize that these proportions should not therefore be associated with any kind of confidence in the predictions, and any interpretation derived from these proportions should be analyzed with caution.

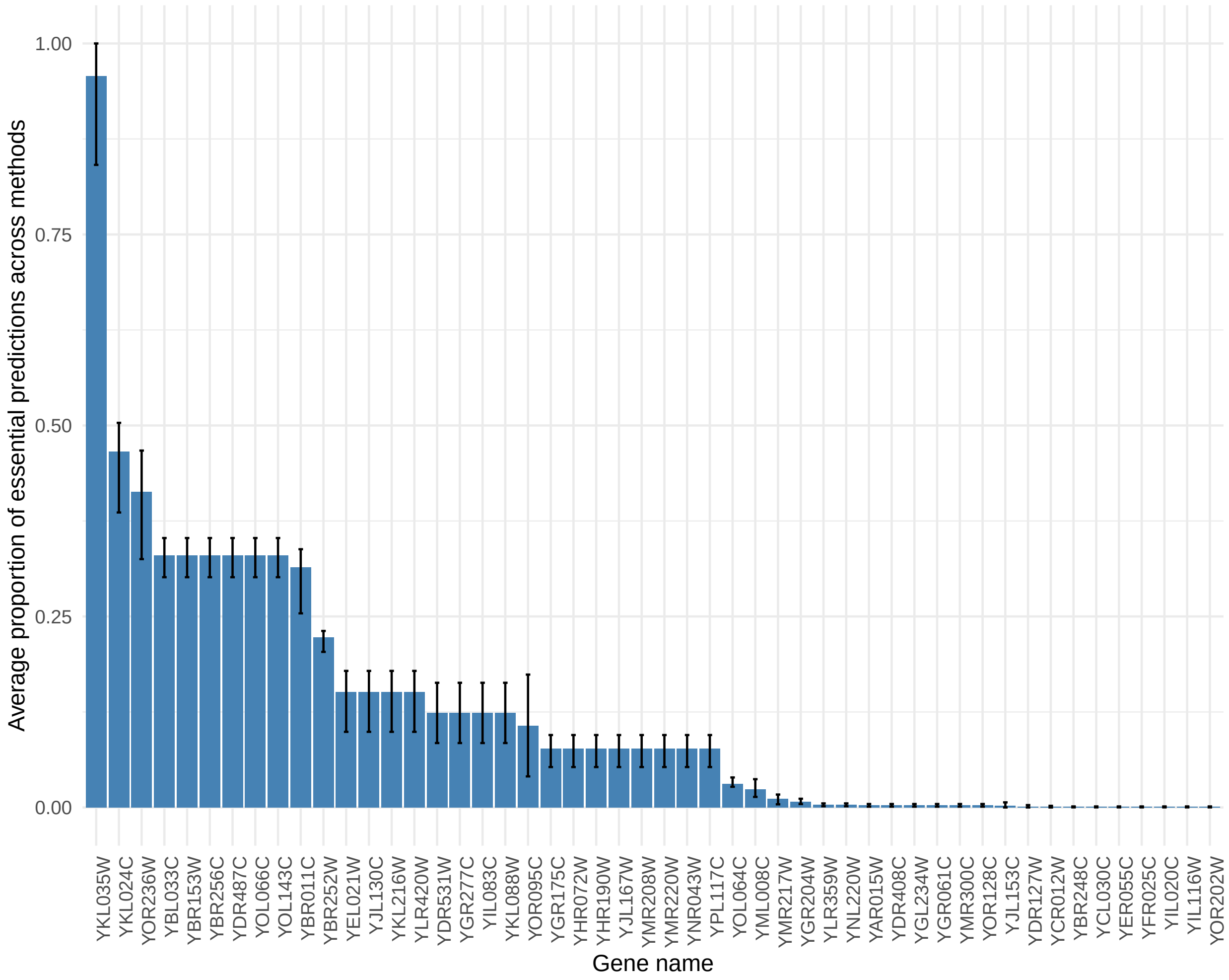

Figure 3: Top 50 most correctly predicted essential genes (after removing the 47 genes that are always correctly classified by all the networks/methods). The Y-axis shows the average proportion of times that each gene was correctly predicted as essential across all networks/methods. Error bars show the differences between methods (minimum and maximum proportion).

Variability in the detection of enriched pathways between the different methods

We calculated how often each pathway was detected as enriched (corrected p-value ≤ 0.05) by any optimal metabolic network for each enumeration method. Figures 4 and 5 show the distribution of the proportion of times that each pathway was enriched for the cell line A375 (threshold [0.10, 0.90] and [0.25, 0.75]). The interval corresponds to the variability between methods (standard deviation of the proportion of networks with enrichments in that pathway by each methods). Same for cell line HL60 (Fig. 6 and 7), cell line K562 (Fig. 8 and 9) and cell line KBM7 (Fig. 10 and 11). As in the case of essential genes, there are differences in the number of times each pathway appears enriched. Again, the interpretation of these proportions should be done with caution, as this may be due to several factors that might not be related to a biological reason. Some

of the causes that may contribute to such variability are the annotations of the metabolic network (some pathways contain more reactions with no gene associations, which potentially increases the number of variations), the objective function or the genes considered for optimization. With respect to the latter, it can be seen that changes in the threshold used to classify genes into highly expressed or lowly expressed greatly affect these proportions. For example, in the A375 cell line, using a gene expression threshold of [0.10, 0.90], the Cholesterol pathway is enriched in the 100% of the solutions, with no differences between methods. However, using a threshold of [0.25, 0.75] (i.e., adding more genes to the highly expressed and lowly expressed sets) decreases this proportion to only 10%. Adding more genes to both sets based on their gene expression levels risks introducing more irrelevant genes in the optimization problem, which can diminish the enrichment of certain pathways compared to others. This means in this case that if the Cholesterol pathway is dysregulated in A375 cells (as it might be the case [3]), using a threshold of [0.25, 0.75] and a single reconstruction will certainly miss this pathway. In this way, enumerating alternative reconstruction decreases the risk of incorrectly ruling out hypothesis that are equally valid for the same reconstruction method and data.

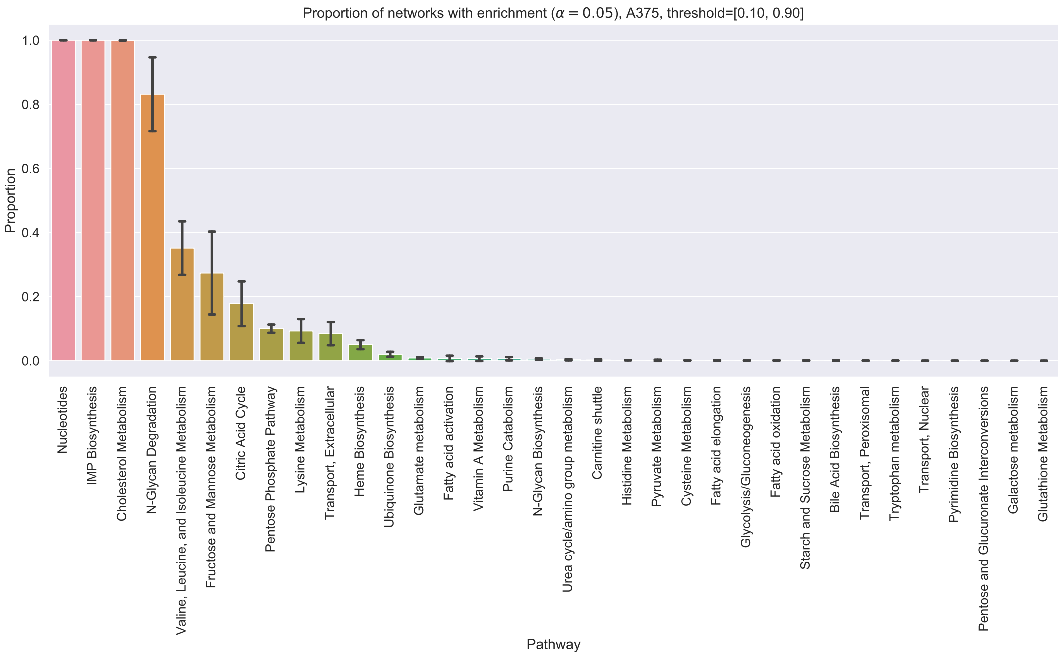

Figure 4: Proportion of times that each pathway was enriched (cell line A375, threshold [0.10, 0.90]). Error bars show the variability (standard deviation of the proportions) due to the enumeration method.

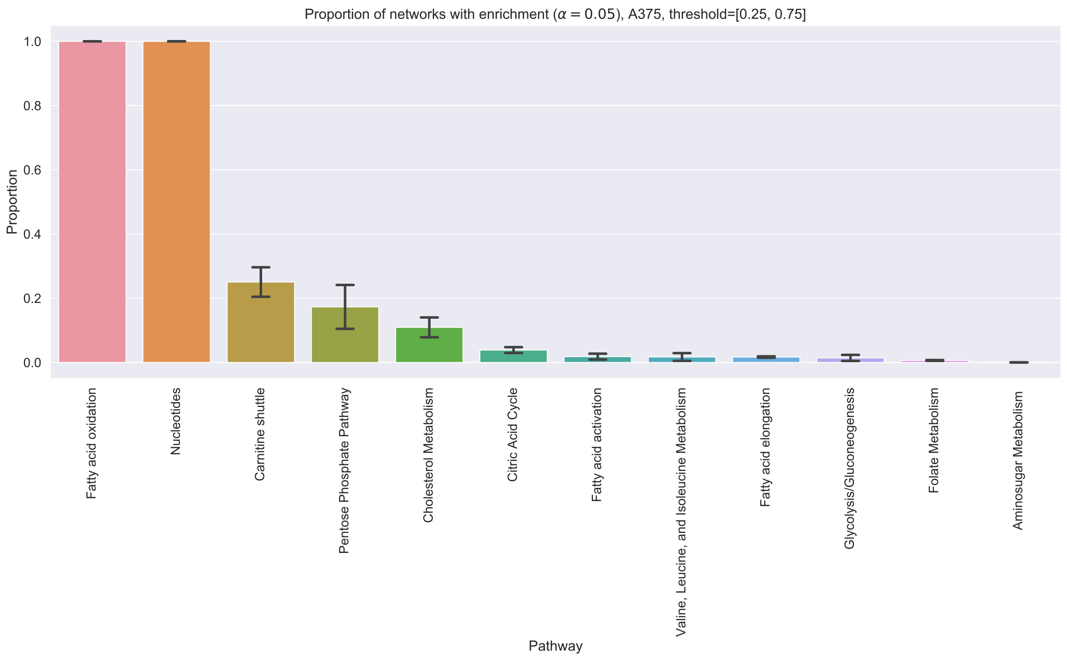

Figure 5: Proportion of times that each pathway was enriched (cell line A375, threshold [0.25, 0.75]). Error bars show the variability (standard deviation of the proportions) due to the enumeration method.

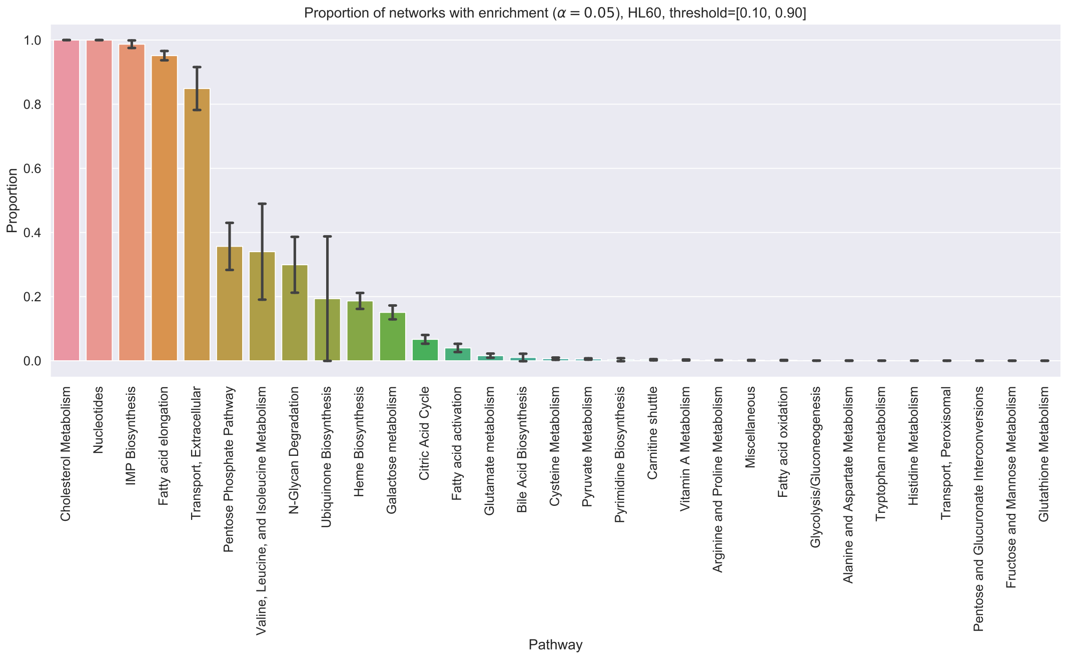

Figure 6: Proportion of times that each pathway was enriched (cell line HL60, threshold [0.10, 0.90]). Error bars show the variability (standard deviation of the proportions) due to the enumeration method.

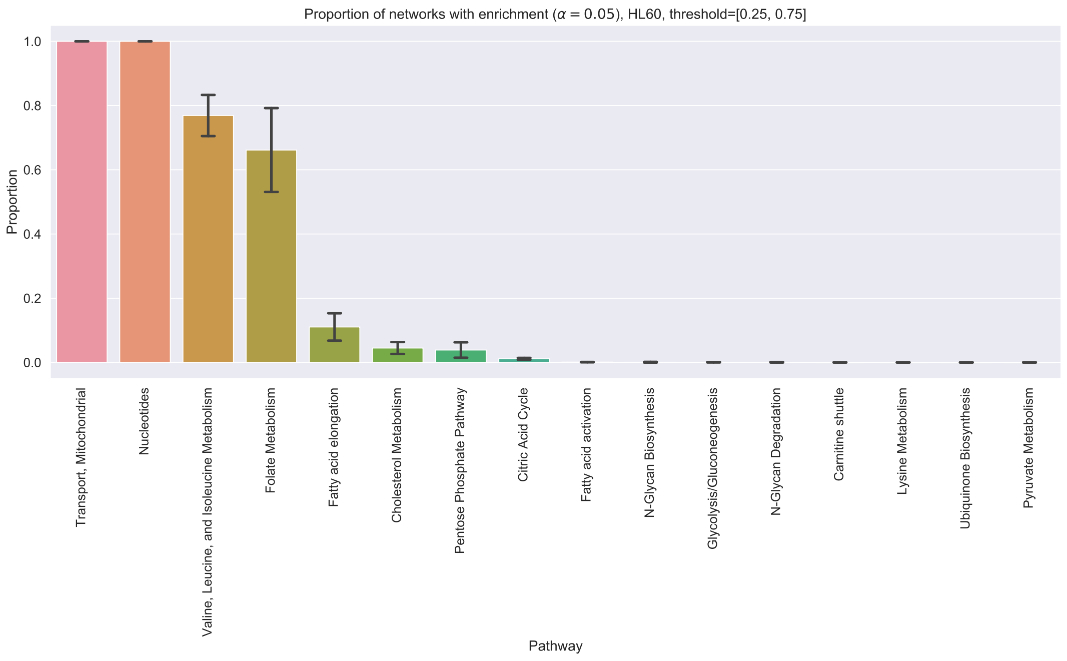

Figure 7: Proportion of times that each pathway was enriched (cell line HL60, threshold [0.25, 0.75]). Error bars show the variability (standard deviation of the proportions) due to the enumeration method.

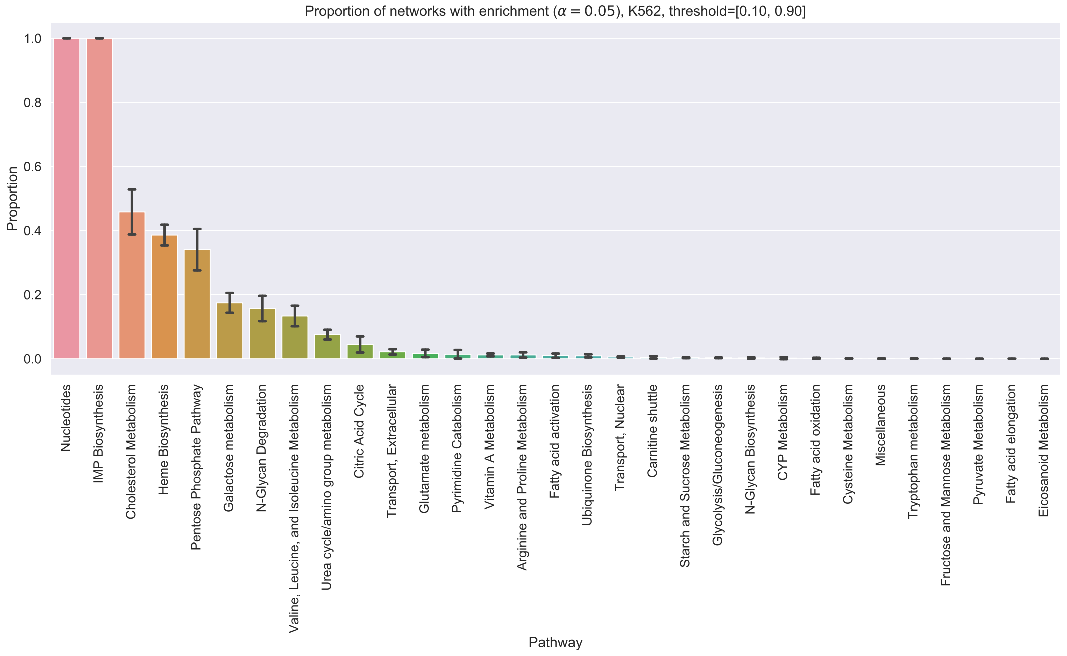

Figure 8: Proportion of times that each pathway was enriched (cell line K562, threshold [0.10, 0.90]). Error bars show the variability (standard deviation of the proportions) due to the enumeration method.

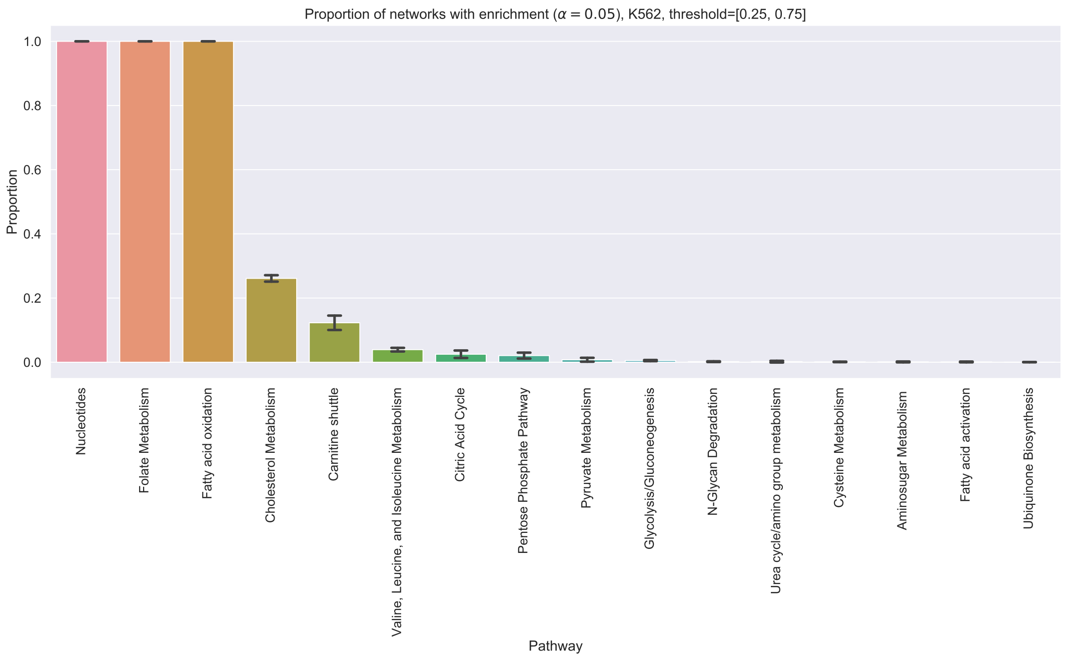

Figure 9: Proportion of times that each pathway was enriched (cell line K562, threshold [0.25, 0.75]). Error bars show the variability (standard deviation of the proportions) due to the enumeration method.

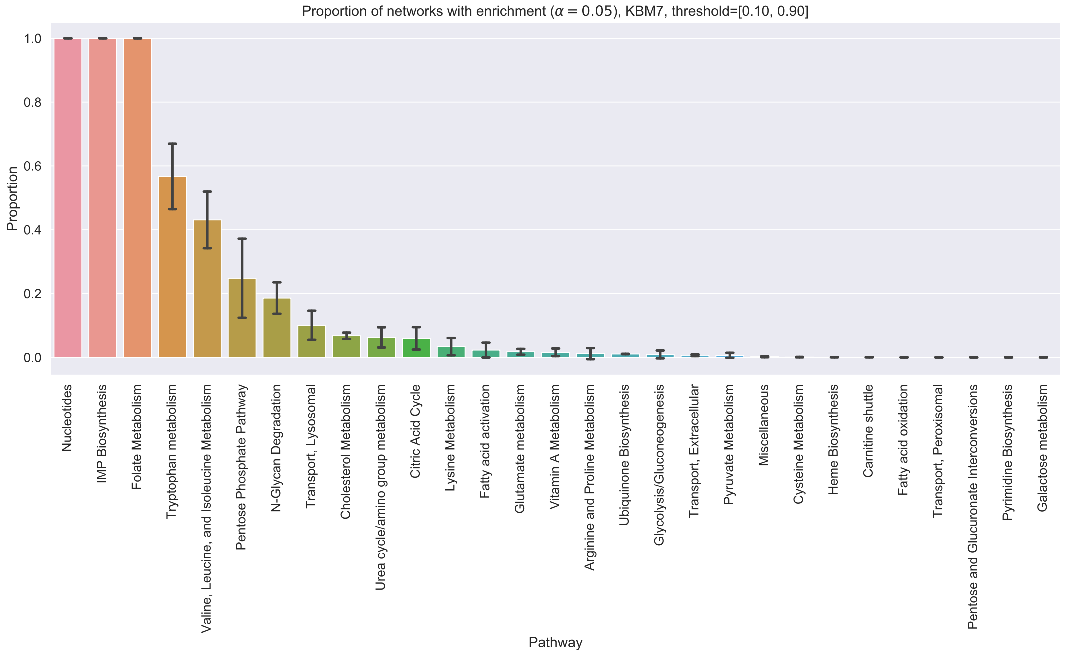

Figure 10: Proportion of times that each pathway was enriched (cell line KBM7, threshold [0.10, 0.90]). Error bars show the variability (standard deviation of the proportions) due to the enumeration method.

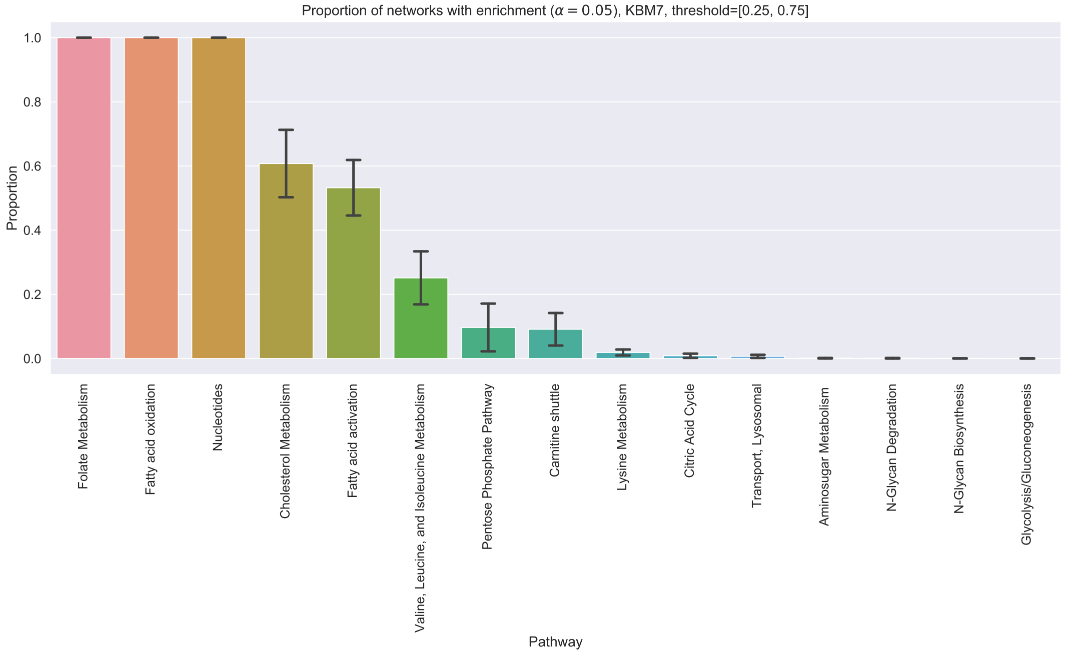

Figure 11: Proportion of times that each pathway was enriched (cell line KBM7, threshold [0.25, 0.75]). Error bars show the variability (standard deviation of the proportions) due to the enumeration method.

References

[1] Heavner BD, Smallbone K, Price ND, Walker LP. Version 6 of the consensus yeast metabolic network refines biochemical coverage and improves model performance. Database. 2013;2013.

[2] Conway JR, Lex A, Gehlenborg N. UpSetR: an R package for the visualization of intersecting sets and their properties. Bioinformatics. 2017;33(18):2938–2940.

[3] Mohammad N, Malvi P, Meena AS, Singh SV, Chaube B, Vannuruswamy G, et al. Cholesterol depletion by methyl-β-cyclodextrin augments tamoxifen induced cell death by enhancing its uptake in melanoma. Molecular Cancer. 2014;13(1):204.
