# Supplementary material for: DEXOM: Diversity-based enumeration of optimal context-specific metabolic networks
Source: PLoS Comput Biol. 2021 Feb 11;17(2):e1008730. doi: 10.1371/journal.pcbi.1008730 (PMC7904180; doi:10.1371/journal.pcbi.1008730)
Supplement: Attachment — Submitted filename: Response.pdf [file pcbi.1008730.s011.pdf]

Authors Response

Manuscript ID: PCOMPBIOL-D-20-01343
Title: DEXOM: Diversity-based enumeration of optimal context-specific metabolic networks

Summary of changes

We would like to thank the reviewers for their valuable suggestions and comments which helped us to further improve the quality of the paper, as well as the *Center for Reproducible Biomedical Modeling* (Referee 1) for their detailed and favourable report about the reproducibility of our work.

This document, on the basis of a careful analysis of the review, presents the responses to each referee as well as the changes that have been made to the original paper. The main changes are:

- We extended our application of `DEXOM` to different human cancer cell lines as an example of eucaryote organism and showing its applicability to large metabolic networks. We used data from [1] to enumerate a set of optimal solutions for each cell line. We also performed downstream analysis of the reconstructions using pathway enrichment to show 1) how the results vary when considering different sets of optimal solutions and 2) which method is able of discovering more variability in terms of different pathways that can be detected as enriched.

- We have also improved the robustness of our experiments by conducting a sensitivity analysis of `DEXOM`. We repeated the experiments for essential gene prediction and we show how result are robust to changes in the parameter d_s which controls the speed at which the algorithm progressively increases the distance of the search of alternative optimal solutions.

- We have rewritten many parts of the article to better present the objectives, the novelties of this work and the advantages of enumerating optimal context-specific metabolic networks.

More especifically, regarding this last point, we have improved the introduction of the paper to make clear the contributions and objectives. We summarized the main objectives and novelties of this work at the end of the introduction:

> - *The analysis and identification of the computational problem involving the diversity-based enumeration of optimal context-specific metabolic network.*
> - *The development of a library (`DEXOM`) including four different methods (Reaction-enum, Icut-enum, Maxdist-enum and Diversity-enum) for the enumeration of optimal context-specific metabolic networks.*

- *An extensive comparison using the different methods under different experimental conditions, showing how variable is usually the space of valid optimal solutions, and comparing the methods in terms of ability to detect these solutions.*
- *The development of an open-source library integrated with COBRA Toolbox 3.0.6 with the different methods for the enumeration of solutions.*

In the initial draft, we referred to the different techniques as `Integer-cut`, `Rxn-enum`, `Maxdist` and `DEXOM`. Those are the techniques we implemented in an unified open-source library (integrated with COBRA Toolbox) that we developed for this paper with the goal of providing them to the community. After reading the reviews, we realized that the contributions were not stated clearly enough. In this work we present four general implementations for the enumeration of context-specific reconstructions. Three of them are adaptations of other techniques with improvements for the particular case of the enumeration of context-specific reconstructions, for which there were no previous implementations and had not been published elsewhere, while the technique we referred as `DEXOM` in the initial draft was a new method that we have designed to improve some of the limitations of the other techniques. Therefore we have decided to refer to the library that combines these techniques as `DEXOM`, more in line with the objective of the paper and the name given to the open-source library that we have developed for this work, and the techniques included in `DEXOM` are now called `Icut-enum` (previously `Integer-cut`), `Reaction-enum` (previously `Rxn-enum`), `Maxdist-enum`, and `Diversity-enum` (previously referred simply as `DEXOM` and `dexom-default` in the script files).

Also, in order to show this approach can be used with other models, we used the four methods to enumerate optimal reconstructions of cancer cells with the human Recon 1 model, and we also included examples of how to reconstruct multiple optimal metabolic networks using DEXOM and the Recon3D model. Examples are included in the code repository[1].

The changes are also highlighted in the additional manuscript provided for that purpose.

[1] Script `scriptExampleCancerReconstructionRecon3D.m` in `https://github.com/MetExplore/dexom/tree/master/src/evaluation`

Response to Referee 2

Q-1.1.1 *Please provide a line numbered version, so reviewers can point out the location of reference easily.*

A-1.1.1 We added line numbers to this new version to facilitate the review.

Q-1.1.2 *How prevalent is the usage of DAG in understanding metabolic networks. Perhaps authors could better highlight and justify their use of DAGs in Introduction.*

A-1.1.1 Directed Acyclic Graphs are commonly used for the analysis of biology networks in general [2]. In our particular case, this model is useful to create toy models with specific properties that we know in advance and help us to define the problem of enumeration. For example, given the number of layers and metabolites and reactions in the DAG model, we can calculate in advance how many optimal solutions we can expect, and thus compare the techniques with a ground truth in an objective manner focusing specifically on the computational problem of enumeration. This is important because although the scope of application is biological, the technique is basically computational and requires a proper computational analysis of the problem under study. We included the following paragraph in the *Methods* section:

> *In order to better analyze the enumeration problem from a computational point of view, we use a Directed Acyclic Graph (DAG) network model. Directed Acyclic Graphs are commonly used for the analysis of biology networks [2]. By representing metabolic networks as DAGs, we can calculate in advance how many optimal solutions we can expect, and thus compare the techniques with a ground truth (i.e., the full set of optimal solutions that exist in a specific DAG) in an objective manner focusing specifically on the computational problem of enumeration. This is important because although the scope of application is biological, the technique is basically computational and requires a proper computational analysis of the problem under study.*

Q-1.1.3 *In regards to TPR and FPR, the authors chose to report the ratios. Could they, please, also show the actual numbers? I am curious how they get influenced from difference between low to high thresholds.*

A-1.1.3 All the results are available both in Matlab files and exported CSV files in the repository[2]. For convenience, we have exported it and included it directly in this document (See Table 1).

Q-1.1.4 *Are Table 1, Figure 7, and Figure 8 coming from the same set of simulations? If yes, could authors please either concise some of this information into a single figure or put some of it in supplementary information?*

A-1.1.4 Yes, the idea is to show not only the results of the ensembles (the aggregated predictions of the networks) but also the predictions of each individual

[2] https://github.com/MetExplore/dexom-evaluation/blob/master/results/yeast/essential_genes/yeast6-evaluation/dexom-yeast6-eval-export-csv.zip

network. We decided to move the Figure 7 and 8 (now S6, S7) to supplementary information as it provides only additional information about the experiment.

Q-1.1.5 *Could the authors, please, provide the link to the method within the paper somewhere as well.*

A-1.1.5 We would like to thank the reviewer for noticing this. We removed the link from the abstract by mistake. We added it back again in the abstract and also in the introduction.

Q-1.2.1 *I want to make sure that by optimal context-specific model they mean, models which maximizes the addition of active and removal of inactive reactions with experimental evidence. Doesn't this definition assume that the output of the thresholding is accurate? Besides global thresholding used in this paper, recent studies have identified better thresholding approaches that generate more accurate models, also some thresholding approaches have enforced biological significance. Have the authors looked at some of these thresholding approaches to see if DEXOM can do better with better thresholds?.*

A-1.2.1 In this work we focus on the problem of enumeration and not in the pre-processing step. The problem of enumerating solutions comes after the problem of selecting which reactions to minimize of maximize. Different methods that uses experimental data to classify the importance of reactions (global thresholds, other techniques such as StanDep, Barcode...) will result in different sets of reactions to preserve or remove from the reconstruction. Regardless of the method chosen, the problem of enumeration persists. This implies that regardless of how good the chosen method is, there could still be multiple different optimal networks (due to the discrete nature of the problem and the imbalance between available constraints and complex topology of the network leading to an undertermined problem) but only one will be obtained by the reconstruction method (e.g. iMAT, Fastcore, ...) without knowing which one or how variable the solutions can be.

In this paper we focus on how to analyze this variability that otherwise is simply not considered and not analyzed. We also show how this variability is important not only for interpretation but also for improving the predictions derived from the extracted sub-networks. Of course, as pointed out by the reviewer, the final quality of the models and ensembles depends also on how good this classification of reactions is, but this is true for all reconstruction methods. That is why we went a little further in our experiments and tried many combinations of thresholds to better appreciate the variability of the results, and how there is always a benefit of recovering multiple solutions (for example, ensembles are always better for predicting essential genes than individual networks, regardless of the threshold used, as shown in the Figures S6 (TPR distribution) and S7 (FPR distribution), where individual networks are worse than ensembles).

We thank the reviewer for bringing up this question because it may have not been sufficiently explained in the first version of the article, although we have not devoted time on this question because it is outside the scope of this work. To better clarify this question, we added a new subsection called "Data pre-processing" explaining this issue, which was not well explained in the original

draft. We also reference StanDep and Barcode as an alternative method to global thresholds.

Q-1.2.2 *A lot of the work on how to measure diversity has been done, i.e. the diversity between models has often been reported using Jaccard similarity (see Fastcore line of papers and papers evaluating context-pecific extraction method). The behavior of this distance metric with reaction active with experimental evidence and those of the models has been shown in these papers. The authors can choose to use Hamming distance, but it becomes difficult for the reader to compare results in those papers with those reported by the authors. Could the authors please provide some justification for their choice to use Hamming distance? Alternative authors could also show that these results could be reproduced using Jaccard similarity.*

A-1.2.2 Both measures are highly correlated, but there is an important reason for which we preferred Hamming distances and not Jaccard distances as a metric to maximize distance between metabolic networks. We analyzed both metrics and we realized that Hamming distance is a better distance for the particular case of this work, that is, for obtaining a diverse set of solutions. We did some experiments to show this[3]. The main reason is that the Hamming distance is a lower bound of the Jaccard distance for binary vectors, i.e., the Jaccard distance between two different solutions is always, at least, their Hamming distance. This means that two solutions with identical Hamming distance may have different Jaccard distances. Thus, maximizing Jaccard distances returns less solutions in general, which goes against the idea of diversity, but we do not find any biological reason to discard solutions that are still different in at least one reaction. The Hamming distance is thus less strict and makes less assumptions about the differences between solutions.

Q-1.2.3 *Robaina-Estevez et al. 2017 found that CORDA, a methods that enforced metabolic functionality rather than the reactions performed better. Does this statement still hold true with DEXOM using gene essentiality as metric?.*

A-1.2.3 There is always and advantage of detecting multiple solutions. A simple example is the following. After enforcing metabolic functionality to the original reconstruction problem, there are three options: 1) there is no feasible reconstruction to this new problem; 2) there is only one metabolic network; or 3) there are many possible solutions to the problem. In general, as we explain in our work, given the combinatorial nature of the problem and the size of the metabolic networks, it is easy to find thousands of optimal solutions. For example, many reconstructions can achieve the same metabolic functions but using alternative pathways. Since metabolic networks are not perfect models, some of these solutions can use pathways that are biologically implausible, and other solutions might be more realistic or more close to what happens in reality. Now, if enumeration is not used, and the method returns only one solutions and we use it to predict genes that are essential, the solution can be good simply by luck. If there is another one that is equally optimal and the method does not return it, the solution that has been provided does not say much about how good the

[3] `https://colab.research.google.com/drive/17Aw_PCu0ER56pfVDn6cv6EDid4UQ4LPL?usp=sharing`

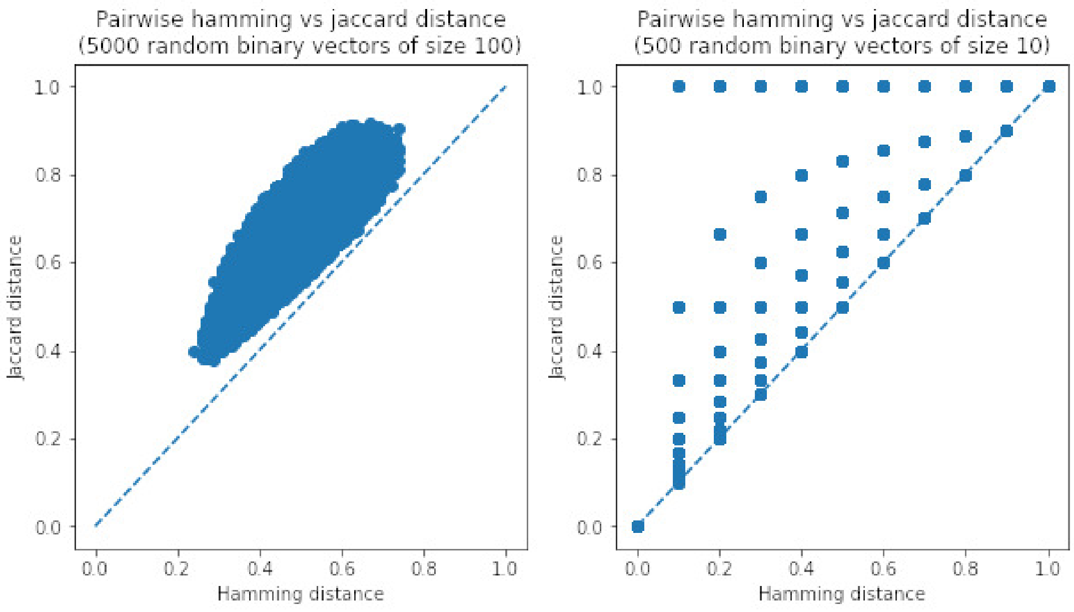

Figure 1: Scatter plot showing the correlation between Hamming distances and Jaccard distances for random binary vectors.

method is for predicting essential genes. What is worse, this affects the reproducibility of the results. The same reconstruction algorithm on another machine can return a solution whose prediction is much worse. Enumerating the space of possible solutions is always better because it allows to know how variable these predictions can be.

Since in this work we propose and compare methods for enumerating solutions, new constraints like these (production/consumption of certain metabolites from certain substrates) can be perfectly added to the MILP problem and use any of the techniques proposed here for enumerating solutions. We do not address the particular question of metabolic functionality because it is not the purpose of this work, and the problem of multiple solutions applies equally in this case as well. So in summary, if there are several metabolic sub-networks that can achieve the same metabolic functionality using different metabolic paths but you capture only one, then you might miss or artificially predict some essential genes.

It is important to note that the methods we propose here are agnostic to the particular reconstruction problem, as long as they can be modeled as MILP problems.

Q-1.2.4 *This is tied to point 4, how does DEXOM do if it is coupled with metabolic functionality.*

A-1.2.4 This answer is similar to the previous one. Enforcing metabolic functionality can be done by adding constraints to the reconstruction problem. What `DEXOM` enables is the enumeration of multiple solutions in a general way, offering different strategies for this. This is factible and can be done by adding more constraints to the reconstruction problem. Nevertheless this does not prevent from the need of doing enumeration.

Enumerating the alternative solutions leaves less things to chance (is the solution obtained the only one or are there more?, perhaps there are others that are better or more plausible that the same reconstruction method can return).

Q-1.2.5 *For improving these types of context-specific models, two major ways have been targeted: (1) extraction method, and (2) thresholding. The authors are dealing with a novel extraction method. As a way to point out if this is the way forward, can the authors compare their TPR/FPR with those achieved from changing the thresholding method.*

A-1.2.5 We would like to emphasize that the methods presented in this work are not extraction methods but generalized algorithms for enumeration or sampling context-specific reconstructions. This means that they can be used with different context specific reconstruction strategies (e.g., to enumerate iMAT-like solutions, Fastcore-like solutions...), enumerate solutions enforcing some specific metabolic functions, or any other context-specific reconstruction objective that can be modeled as a MILP problem. The advantage of this is that we do not propose a specific method of reconstruction, since the objective is different for each problem. For example, in cancer cells it is difficult to follow a method that enforces specific metabolic functions because they are not well known. We offer techniques that allow the enumeration of alternative solutions, whatever the objective to be solved. We believe that this is the way forward, since it is complementary also to other thresholding methods or different reconstruction objectives. As explained in A-1.2.3, better methods of data-preprocessing can be used to improve the quality of the reconstructions. We consider that the evaluation of different thresholding methods, although interesting, would be the object of a different study.

Q-1.2.6 *Different algorithms are enforcing different interepretations of how context-specific metabolic networks are organized. Whether the interpretation is true for only yeast, single-celled organisms, eukaryotes, or fungi, or other organisms too. Yeast is a single cell organism which can be grown in the lab relatively easily compared to mammalian cells. Extensive comparison data and models are available if DEXOM was applied to human tissues, mouse, and/or cancer cell lines. Could the authors show that DEXOM can be extrapolated to other organisms and/or datasets?*

A-1.2.6 We acknowledge that this is an important issue, so we extended our results to perform enumeration of context-specific reconstructions of four human cancer cell lines, using data and models from [1] (Recon 1 and RNA-seq data), generating in total around 67,400 reconstructions. We used pathway enrichment to test for overrepresentation of reactions in cancer cells, and we show how variable results can be. We compare the ability of each technique to discover alternative hypotheses of the metabolic state. In addition to this, we added an example to the repository to show how to perform enumeration of optimal solutions using the Recon 3D model.

Response to Referee 3

Q-2.1 *Although the diversity seems to be sampled best by DEXOM as visually shown by UMAP based dimension reduction, the advantage of the improved metrics*

seems to be not always the case as shown in panels E and F of Fig. 5 and S1-S3 and depends to a large quantity on the sampled networks (cf. e.g. S3 against S2 and S1). Hence, it appears that it cannot be guaranteed that DEXOM outperforms Maxdist with respect to the given metrics. In addition, as shown in Table 1 and Figure 7 and 8, the advantage of using DEXOM over Rxn-enum for finding essential genes in a given network is not substantial. Specifically the increased TPR is often achieved at the price of an increased FPR, while the differences in TPR and FPR for Rxn-enum and DEXOM are generally very low. This seems to render Rxn-enum the better algorithm for identifying gene essentiality due to its low runtime (compared in Fig. 9), despite providing only a restricted and not diverse set of optimal networks. In summary, the advantages of using and applying DEXOM are not obvious and the authors need to justify why DEXOM should be used instead of Rxn-enum (for finding essential genes) or Maxdist (for computing a diverse set alternative optimal networks for further downstream analysis like pathway enrichment).

A-2.2 We thank the reviewer for his comments, as we have realized two things: 1) the contributions of the paper were not clearly enough stated; and 2) we needed to improve the discussion regarding the advantages and disadvantages of the four methods.

One of the contributions of this paper is precisely to offer different techniques to enumerate context-specific reconstructions, and compare them. Some of the techniques presented in this article (Reaction-enum, Icut-enum and Maxdist-enum) are inspired in other techniques, improved and adapted to the particular case of sampling diverse solutions, and are not published elsewhere. No implementations are available either. More specifically, Reaction-enum as a way to generate alternative solutions was for the first time used within our group ([3]) but many questions remained without answer (Is this technique able to enumerate the whole space of possible solutions? Is there any other way to generate alternative solutions? How useful are these alternative solutions? Could it be improved?). We try to answer all these questions in our paper. Also, as far as we know, integer-cuts were not explored as a way to generate alternative context-specific metabolic networks, even though it was used before in Operations Research as a general mechanism to enumerate optimal solutions in MILPs. We thus adapted this technique as well to this specific problem and analyze its limits and advantages. The idea of maximizing dissimilarity was used before for the enumeration of solutions in general [4, 5] and also in context specific reconstruction [6]. However, in the paper we discuss problems that make this technique not practical and we improve it for the enumeration of context-specific reconstructions. Also, no usable algorithm was available to enumerate context-specific reconstructions, so we developed the modified version and included it in our library. During this process, we discovered that all these techniques, although useful, had also limitations, and we developed a new one with the idea of improving the diversity of the solutions obtained.

The contributions therefore are the different techniques (and their corresponding usable implementations in Matlab) and the extensive comparison that allows to decide which techniques are more interesting for different practical scenarios. We extended also the results section as suggested by the reviewer in order to show another practical advantage of a more diverse set of solutions,

using pathway enrichment as a downstream analysis of human cancer cells.

As the reviewer points out, depending on a particular case, some technique might be preferable. For example, if the only purpose is to do in-silico predictions of essential genes, the Reaction-enum technique might be enough (depending on the computation time) since in practice we did not find large differences. In any case, we observed in general an advantage of using `Diversity-enum`. To make sure that this advantage was not simply due to chance, we have done a sensitivity analysis of the d_s parameter used in the `Diversity-enum` method, repeating the enumeration of solutions and the prediction of essential genes, and we observed similar results (Figure S5).

In the new experiment using four different human cancer cell lines from [1], we used pathway enrichment as a downstream analysis of the reconstructed networks, and again we observed that `Diversity-enum` was the technique that found more extreme results in terms of enrichment in all cases except in one case —out of eight cases— (cell line K562 with thresholds [0.25, 0.75]) in which `Reaction-enum` found more alternative solutions enriched for other pathways.

In terms of diversity of solutions, all the experiments with `Diversity-enum` were done with $d_s = 0.995$, which we found that in practice presents a good behaviour for medium size metabolic networks, but this parameter can be adjusted to increase faster the distance of the new solutions. Since we have not adjusted the parameter case by case, there are situations like those in the figure mentioned by the reviewer where the rate of growth of the distances is slower and does not reach (or exceed) `Maxdist-enum` for the fixed number iterations used. Using the default value $d_s = 0.995$ means that after computing the initial set of solutions, (e.g., iteration 140 after the initial set of solutions was calculated), the expected distance of the next solution with respect the previous solution would be $1 - 0.995^{140} \approx 0.50$ of the maximum possible distance. At iteration 1,000, the expected distance of the next optimal solution would be $1 - 0.995^{1000} \approx 0.99$. Note that a value of 1.0 ($d_s = 0$) means that the distance of the next solution would be the maximal distance to the previous solution, which is what `Maxdist-enum` does. In this way, `Diversity-enum` method can be seen as a generalization of `Maxdist`.

In summary, we have found that `Diversity-enum` is a versatile method that is able to produce in general a diverse set of solutions, and to generate sets of solutions with biological importance in terms of prediction (essential genes) and interpretation (pathway enrichment). We agree with the reviewer that depending on the purpose of the analysis, the other methods can perform similarly and this is also a contribution derived from our work: to provide the different methods and a extensive comparison so that the users can also choose the ones which is the most adapted for their analysis.

We have made many changes (highlighted in the manuscript) to explain these things better (L.134-171 in the Introduction; L.730-738, L.761-763, L.832-851, L.907-916 in the Results section; and L.982-994 in the Discussion, lines referring the highlighted manuscript)

Q-2.2 *No usable DEXOM algorithm is provided. This work would greatly benefit from having a ready made software code available that based on any given SBML model allows to compute a diverse set of alternative optimal metabolic models.*

A-2.2 We completely agree with the reviewer that providing the implementation of the methods is important. This is why we provided all the code and data, including the scripts to reproduce the results and figures, with the very first draft of this work. We have also invested time in making the library integrated with COBRA Toolbox so it can be easily used by other researchers. We have also submitted the code to the Reproducibility Modelling pilot of PLOS Computational Biology which confirmed that the experiments are reproducible.

We apologize for not including the link in the main paper, it was removed from the abstract by mistake and it was only included in the first pages of the draft before the main paper. We added back the link to the code in this new draft, both in the abstract and at the end of the introduction. The code is available at `https://github.com/MetExplore/dexom`.

Q-2.3 *Only one model and one dataset on yeast are used for demonstrating and comparing the performance of the proposed algorithm and this only for gene essentiality analysis. Although mentioned in the introduction as potential use case the authors did not include any model analysis to investigate diseases in e.g. human, presumably because human reconstructions like Recon3D possess a large number of reactions, which make it computationally demanding to compute optimal solutions. Nevertheless, it remains unknown how DEXOM performs on other data. At least one further data set analysis, potentially with a different aim than identifying gene essentiality (e.g. enriched function) of another organism would greatly improve the trust in DEXOM, if it can be shown that it outperforms competing methods.*

A-2.3 We agree about the importance of applying the methods to other organisms and for different purposes. As mentioned before, we extended the experimental results by performing enumeration of context-specific metabolic networks of four different cancer cell lines, using the data and model (Recon 1) provided by the authors in [1]. Downstream analysis using pathway enrichment shows that `Diversity-enum` is the technique that identifies more different solutions with enrichments in diverse pathway. In addition, we show that the enumeration methods can be used with different models. We added an example in the GitHub repository[4].

Q-2.4 *The authors do not discuss the virtually equal quality of Rxn-enum vs DEXOM for gene essentiality analysis. Furthermore, TPR and FPR are not given for the alternative sets presented in S1-S3, which show much different hamming and nearest neighbor values, which presumably influence achievable TPR and FPR values. Code and solutions are not given to allow recapitulating the analysis.*

A-2.4 We improved the paper to discuss this (see A-2.3) and we also repeated the analysis for different values of the d_s parameter. Regarding the TPR and FPR of figures S1-S3, we did not do that because we used random sets of reactions (R_H and R_L were randomly selected with fixed sizes of 60, 80, 100 and 120 reactions) for the reconstruction, and we did not use at this point any gene expression data for yeast under aerobic conditions. The goal of this experiment is similar to the goal of the enumeration using the DAG network, but using a biologically

[4]Script `scriptExampleCancerReconstructionRecon3D.m` in `https://github.com/MetExplore/dexom/tree/master/src/evaluation`

relevant model with real structure (however in this example it is not possible to know the full space of optimal solutions in advance). We decided to do this using random sets to generate more variable scenarios, but there is no connection with the experimental data used for the evaluation of essential genes. Regarding the similar results obtained with both methods, this could be explained by the fact that both methods explore better than the others the space of optimal solutions that come from single variations in reactions. Since the simulation of essential genes is based on simulating knockouts in the reactions associated with the genes, it is likely that the space of alternative solutions relevant to this problem will be in that area of the solution space and not in other parts. This idea is also supported by the fact that results do not change a lot by varying the d_s parameters (Figure S5), but there is still an advantage for the `Diversity-enum` method, since it progressively expands the initial set of solutions and is able to discover more solutions than `Reaction-enum` that are closer to the initial ones, increasing the probability of detecting alternative solutions relevant to the problem.

We added the following paragraph (L.828-847) at the end of the subsection *Prediction of essential genes using ensembles is highly dependent on the strategy used for enumeration* discussing this:

> *In order to test whether the distance parameter d_s has some strong impact on the results obtained with `Diversity-enum` method, we repeated the same experiment with parameter values $d_s = 0.990$ and $d_s = 0.999$ (See supplementary figure S5). In both cases, the results obtained are very similar to these results obtained with the default parameter $d_s = 0.995$. This result suggest that most relevant solutions to the problem of prediction of essential genes are concentrated in the same region of the space of optimal solutions that is explored by both `Reaction-enum` and `Diversity-enum`. This space corresponds to the alternative solutions generated by modifying the constraints of single reactions in the networks (forcing the inclusion or knocking-out the reaction). Since the simulation of essential genes is based on simulating knockouts in the reactions associated with the genes, it is likely that most of the essential genes can be predicted in some of the optimal networks resulting from those variations in single reactions. However, there is still an advantage in using `Diversity-enum`, since it expands the initial set of solutions and is able to search for many more than the other technique is not capable of, increasing the probability of detecting more relevant reconstructions.*

Q-2.5 *Throughout the manuscript preciseness is missing. Parameters and functions of the presented algorithm are not described (e.g. exp(a,b) = a^b is not explained). Figures are not described well (subpanel description are not given in captions and are most often not referenced in the main text). Another example is the definition and use of thresholds used in table 1, which need to be briefly introduced in the main text (e.g. at page 20).*

A-2.5 We have improved the quality of the manuscript by adding more detailed explanations in the legend of the figures and by better explaining the parameters used in this work. Also, we added a subsection in *Methods* explaining the thresholds.

Q-2.6 *A major rewrite is recommended, as much of the methods section is recapitulating methods that are published elsewhere. Instead the focus should be on the DEXOM description, while the extensive text body on alternate methods (essentially page 4-13) leading towards DEXOM should be shortened dramatically in the main text. The detailed description would be suitable for a supplementary text that can be referenced in the methods.*

A-2.6 As we explain in A-2.2, all the methods presented in this paper are novel implementations (`Diversity-enum`) or improvements/adaptations of other techniques which have never been published as techniques for the enumeration of context-specific reconstruction (not at least in the form used in this work and with the modifications and improvements implemented). We consider that all the techniques are contributions of this work and thus its description in the main text is important to understand the whole article. The techniques that are inspired in other ideas are cited and properly mentioned in the manuscript.

Minor concerns

- page 3: 10.1016/j.cels.2017.01.010 and https://doi.org/10.1371/journal.pcbi.1003580 are two references that should be mentioned here as they show performances of methods tailored towards computing context specific metabolic models.
  - We thank the reviewer #2 for this suggestion. We have included both references in the introduction. We have also used data regarding the human cancer cell lines from 10.1016/j.cels.2017.01.010 to extend our results.
- page 8: "... extensively exploited in commercial solvers such as IBM CPLEX and Gurobi"- both provide academic free academic licenses, which would be great to mention.
  - We added a footnote mentioning this and providing the links to the web pages.
- page 14: "It starts computing an initial set of solutions using the Rxn-enum method avoiding duplicated solutions. This guarantees that single variations of reactions across all pathways are explored." - This is confusing, as the initial set of solutions will not necessarily include variations across pathways, unless the initial set size is sufficiently high.
  - We clarified this issue:

... as long as this initial set of solutions is large enough (i.e., all reactions of the network are traversed to generate alternative solutions) (page 14).

- page 14: "Using a ds value close to one (e.g. ds = 0.99), the search concentrates at the beginning with more probability in the close vicinity of the selected solution" - Why should it be desirable to start with similar solutions as most diverse solutions are the goal? Of course the formulation allows to start with more distant solutions. It should be made clear here, why similar solutions might be desirable to be computed first.

  – We explained the reason why we finally adopted this strategy and not the opposite one:

    Some preliminary experiments that we carried out suggest that it is preferable to start with the initial set of solutions using the `Reaction-enum` *method and expand it by progressively looking for more distant solutions, rather than the other way around. The reason is that if we start with the most extreme solutions, as we progressively decrease the distance, the effect we get is to explore solutions that are closer to each other but still located in the extremes of the space, and still far from the initial solutions.*

- page 15: "For example, in Saccharomyces Cerevisiae, gene ARG2, which encodes glutamate N-acetyltransferase —a mitochondrial enzyme that catalyzes the first step in the biosynthesis of the arginine— is essential only in the absence of arginine in the medium." - It should be made clear that this is might be a general theme in arginine metabolism, not just in S cerevisae (lowercase for cerevisiae)

  – We rephrased it to:

    For example, the gene ARG2, which encodes glutamate N-acetyltransferase —a mitochondrial enzyme that catalyzes the first step in the biosynthesis of the arginine— is annotated as a essential gene in Saccharomyces cerevisiae[5] *only in the absence of arginine in the medium.*

- page 15: COBRA toolbox reference should be cited here and elsewhere. Also Yeast 6 is not referenced everywhere (e.g. page 16)

  – We added the missing references.

- page 17: "The grey points correspond to the 1,024 optimal solutions of the ground truth." - This is very unclear at this point. Please improve clarity of description of the figures here and elsewhere.

[5] `https://www.yeastgenome.org/locus/S000003607`

- We rephrased to:

  The grey points correspond to the total of 1,024 optimal solutions that exist for this example.

- page 17: "The Maxdist method shows at the beginning of the search the largest distance, since the solutions are generated by finding extreme differences. After an initial set of 25 optimal solutions, the average distance stops increasing. This is something to expect since the most distant solutions are usually discovered at the beginning of the search." - The question arises, whether having the first most diverse networks with Maxdist is sufficient and fast enough as both hamming and nearest neighbor distance are high. This should be analysed, mentioned and discussed.

  - As we show in the Results section, this depends on each particular case. We rephrased the paragraph to explain this:

    After an initial set of 25 optimal solutions, the average distance stops increasing, but the average nearest neighbor distance continues to decrease. This means that the most distant solutions are discovered at the beginning of the search and then there is less and less distance between new found solutions, something to expect given the reduced number of possible solutions in this example. Whether this small number of solutions (around 2% of the total number of equally valid solutions) is sufficient or not will depend on each particular case (for example, it can be enough to show an example of how extreme results can be in terms of different sets of reactions, but not enough to construct a good ensemble for the prediction of essential genes, as shown in the next subsection)

- page 19: "After DEXOM generates an initial set of around 600 solutions, both the average distance and the average nearest neighbor distance start to grow surpassing the other methods" - It is surprising that there is not a gradual, but sudden improvement by DEXOM (also in Fig. 4 for the DAG model). The authors should explain or at least hypothesize on why this is happening abruptly and not monotoically increasing for both hamming and neirest neighboor distance. Regarding particularly investigation in the supplement it appears that for low numbers of sample networks, Maxdist is the better method in terms metric performances. Again, the authors should discuss this point.

  - There are different factors that affect this, but it can be controlled by adjusting the parameter d_s depending on the particular purposes. We added the following paragraph:

    The rate at which this distance grows depends on several factors, including: the distance between the initial solutions, the space of

possible solutions, and the parameter d_s, which controls the rate at which the distance of new solutions increases.

- page 20: Since [38] lists gene expression under low oxygen levels (at most 2.8%), the authors should discuss briefly, whether there is a difference to be expected for higher oxygen levels and how this might affect gene essentiality.

  – We did not analyze this case because the genes that are essential under anaerobic conditions are not well known/annotated. As far as we know, most of the data (Yeast Deletion Project) have been collected under aerobic conditions. Therefore, it is difficult to know if the predictions in this case are correct or not.

- page 20: "Thus, in total, we generated 16 ensembles per method, one for each threshold." - Thresholds are not defined.

  – We have included a sub-section called "*Data pre-processing*" where we explain the thresholds.

- Table 1 would be much easier to investigate as bar plot with four bars (4 methods) for each threshold configuration

  – We thank the reviewer for this idea, although this is somewhat subjective. In our case it is clearer to see the results as shown in Fig. 7, which collect all the data in the table. This ROC plot is commonly used to show the results of classifiers in an accurate way.

- methods and results/discussion should be clearly separated (e.g. machine configuration mentioned on page 24 is Methods content)

  – We moved these details to Supporting Information.

References

[1] Opdam S, Richelle A, Kellman B, Li S, Zielinski DC, Lewis NE. A systematic evaluation of methods for tailoring genome-scale metabolic models. Cell systems. 2017;4(3):318–329.

[2] Junker BH, Schreiber F. Analysis of biological networks. vol. 2. John Wiley & Sons; 2011.

[3] Poupin N, Corlu A, Cabaton NJ, Dubois-Pot-Schneider H, Canlet C, Person E, et al. Large-Scale Modeling Approach Reveals Functional Metabolic Shifts during Hepatic Differentiation. Journal of proteome research. 2018;18(1):204–216.

[4] Danna E, Fenelon M, Gu Z, Wunderling R. Generating multiple solutions for mixed integer programming problems. In: International Conference on Integer Programming and Combinatorial Optimization. Springer; 2007. p. 280–294.

[5] Greistorfer P, Løkketangen A, Voß S, Woodruff DL. Experiments concerning sequential versus simultaneous maximization of objective function and distance. Journal of Heuristics. 2008;14(6):613–625.

[6] Robaina-Estévez S, Nikoloski Z. On the effects of alternative optima in context-specific metabolic model predictions. PLoS computational biology. 2017;13(5):e1005568.

**Threshold**	**Method**	**TPR**	**FPR**	**TP**	**FP**	**TN**	**FN**
[0.10, 0.90]	Diversity-enum	0.7234	0.1264	136	90	622	52
[0.10, 0.90]	Reaction-enum	0.7181	0.1053	135	75	637	53
[0.10, 0.90]	Maxdist-enum	0.4255	0.0730	80	52	660	108
[0.10, 0.90]	Icut-enum	0.4681	0.0815	88	58	654	100
[0.10, 0.85]	Diversity-enum	0.6755	0.0871	127	62	650	61
[0.10, 0.85]	Reaction-enum	0.6649	0.0829	125	59	653	63
[0.10, 0.85]	Maxdist-enum	0.4521	0.0674	85	48	664	103
[0.10, 0.85]	Icut-enum	0.3617	0.0604	68	43	669	120
[0.10, 0.80]	Diversity-enum	0.7128	0.0688	134	49	663	54
[0.10, 0.80]	Reaction-enum	0.7128	0.0716	134	51	661	54
[0.10, 0.80]	Maxdist-enum	0.4149	0.0379	78	27	685	110
[0.10, 0.80]	Icut-enum	0.4096	0.0534	77	38	674	111
[0.10, 0.75]	Diversity-enum	0.6649	0.0843	125	60	652	63
[0.10, 0.75]	Reaction-enum	0.6649	0.0744	125	53	659	63
[0.10, 0.75]	Maxdist-enum	0.4096	0.0548	77	39	673	111
[0.10, 0.75]	Icut-enum	0.3723	0.0548	70	39	673	118
[0.15, 0.90]	Diversity-enum	0.7287	0.1053	137	75	637	51
[0.15, 0.90]	Reaction-enum	0.7234	0.1096	136	78	634	52
[0.15, 0.90]	Maxdist-enum	0.4681	0.0758	88	54	658	100
[0.15, 0.90]	Icut-enum	0.4628	0.0983	87	70	642	101
[0.15, 0.85]	Diversity-enum	0.7128	0.0829	134	59	653	54
[0.15, 0.85]	Reaction-enum	0.7128	0.0576	134	41	671	54
[0.15, 0.85]	Maxdist-enum	0.5000	0.0927	94	66	646	94
[0.15, 0.85]	Icut-enum	0.3617	0.0576	68	41	671	120
[0.15, 0.80]	Diversity-enum	0.7021	0.0885	132	63	649	56
[0.15, 0.80]	Reaction-enum	0.7021	0.0815	132	58	654	56
[0.15, 0.80]	Maxdist-enum	0.3989	0.0323	75	23	689	113
[0.15, 0.80]	Icut-enum	0.3670	0.0548	69	39	673	119
[0.15, 0.75]	Diversity-enum	0.6649	0.0506	125	36	676	63
[0.15, 0.75]	Reaction-enum	0.6649	0.0801	125	57	655	63
[0.15, 0.75]	Maxdist-enum	0.4309	0.0534	81	38	674	107
[0.15, 0.75]	Icut-enum	0.4043	0.0562	76	40	672	112
[0.20, 0.90]	Diversity-enum	0.7181	0.1194	135	85	627	53
[0.20, 0.90]	Reaction-enum	0.7128	0.1025	134	73	639	54
[0.20, 0.90]	Maxdist-enum	0.4734	0.0969	89	69	643	99
[0.20, 0.90]	Icut-enum	0.4628	0.0576	87	41	671	101
[0.20, 0.85]	Diversity-enum	0.6649	0.0927	125	66	646	63
[0.20, 0.85]	Reaction-enum	0.6649	0.0843	125	60	652	63
[0.20, 0.85]	Maxdist-enum	0.5053	0.0702	95	50	662	93
[0.20, 0.85]	Icut-enum	0.4096	0.0365	77	26	686	111
[0.20, 0.80]	Diversity-enum	0.6755	0.0520	127	37	675	61
[0.20, 0.80]	Reaction-enum	0.6596	0.0716	124	51	661	64
[0.20, 0.80]	Maxdist-enum	0.4521	0.0337	85	24	688	103
[0.20, 0.80]	Icut-enum	0.3670	0.0562	69	40	672	119
[0.20, 0.75]	Diversity-enum	0.6702	0.059	126	42	670	62
[0.20, 0.75]	Reaction-enum	0.6702	0.0520	126	37	675	62
[0.20, 0.75]	Maxdist-enum	0.4096	0.0632	77	45	667	111
[0.20, 0.75]	Icut-enum	0.3670	0.0534	69	38	674	119
[0.25, 0.90]	Diversity-enum	0.7713	0.1334	145	95	617	43
[0.25, 0.90]	Reaction-enum	0.7340	0.1194	138	85	627	50
[0.25, 0.90]	Maxdist-enum	0.5213	0.1067	98	76	636	90
[0.25, 0.90]	Icut-enum	0.4787	0.0913	90	65	647	98
[0.25, 0.85]	Diversity-enum	0.6862	0.0885	129	63	649	59
[0.25, 0.85]	Reaction-enum	0.6649	0.0843	125	60	652	63
[0.25, 0.85]	Maxdist-enum	0.4574	0.0730	86	52	660	102
[0.25, 0.85]	Icut-enum	0.4202	0.0590	79	42	670	109
[0.25, 0.80]	Diversity-enum	0.6702	0.0871	126	62	650	62
[0.25, 0.80]	Reaction-enum	0.6702	0.0576	126	41	671	62
[0.25, 0.80]	Maxdist-enum	0.4096	0.0590	77	42	670	111
[0.25, 0.80]	Icut-enum	0.4521	0.0534	85	38	674	103
[0.25, 0.75]	Diversity-enum	0.6755	0.0843	127	60	652	61
[0.25, 0.75]	Reaction-enum	0.6702	0.0801	126	57	655	62
[0.25, 0.75]	Maxdist-enum	0.4096	0.0604	77	43	669	111
[0.25, 0.75]	Icut-enum	0.4096	0.0548	77	39	673	111

Table 1: Detailed results showing the True Positives (TP), True Negatives (TN), False Positives (FP) and False Negatives (FN) of the evaluation of essential gene prediction in yeast.
