# Supplementary material for: DEXOM: Diversity-based enumeration of optimal context-specific metabolic networks
Source: PLoS Comput Biol. 2021 Feb 11;17(2):e1008730. doi: 10.1371/journal.pcbi.1008730 (PMC7904180; doi:10.1371/journal.pcbi.1008730)
Supplement: Attachment — Submitted filename: Response_R2.pdf [file pcbi.1008730.s013.pdf]

Authors Response

Manuscript ID: PCOMPBIOL-D-20-01343R1
Title: DEXOM: Diversity-based enumeration of optimal context-specific metabolic networks

Summary of changes

We would like to thank again the reviewers for their helpful comments on our new version of the manuscript. Based on their reviews, we have improved the quality of the work by including more details about the results. In summary, the main changes are:

- We analyzed the differences in the predictions of essential genes between the different methods. We added a new section in the supplementary file (S1 Appendix) called *"Distribution of predicted essential genes among methods"*, where we show the intersection of correctly predicted genes among methods, and the proportion of times each gene was correctly predicted as essential.

- We have also performed a similar analysis for the pathway enrichment in cancer cells. We added a new section in the supplementary file (S1 Appendix) called *"Variability in the detection of enriched pathways between the different methods"* where we show for each cell line and threshold the proportion of times each pathway was enriched as well as the variability between the different enumeration methods.

Response to Referee 2

Q-2.1 *Why is there a need for 4 different approaches which need to be under one umbrella? Why can't just the best method prevail? Are there any advantages of Icut-enum and Maxdist-enum? These two have very poor accuracy and why do they need to be included in the paper or in the toolbox. Can the authors show, systemically, cases where enumeration using one method is better than other. The authors can do this by finding which essential genes are captured differentially among networks calculated using different methods. I think this is an important benchmarking step for the authors to justify the need of multiple enumeration methods within this toolbox for context-specific networks.*

A-2.1 As suggested by the reviewer, we have included a plot (in the supplementary pdf, S1 Appendix) showing the intersection of the true positive essential genes predicted by the four methods. In the new section "Distribution of predicted essential genes among methods" we show how there are some genes that are captured by some methods but not by others (we have included this figure here for convenience, see Figure 1). Thus, even though `Reaction-enum` and `Diversity-enum` performed better, there still exist differences among methods. For example, `Icut-enum` method detected one essential gene not predicted by any of the other methods.

We have begun this work in response to the lack of methods and proper analysis of the variability in the reconstruction of context-specific networks. This led us to explore different strategies, including basic approaches like adding integer cuts to discard solutions, as a simple strategy that can be implemented on top of any MILP solver. We included all the methods because 1) we did not know a priori which method would perform better; 2) we discovered differences that might be worth to further explore in other research works (essential genes only detected by `Icut-enum` or pathways detected as enriched only by `maxdist-enum`); and 3) the methods could be useful for other purposes, even for purposes that we are not aware of right now, and they could help others to improve/reuse them for their research.

Q-2.2 *To me it appears Rxn-enum and Diversity-enum have similar accuracy. Diversity-enum and Maxdist give the most diversity within the models and they both take a long time to run. So authors generated a method which produces a diverse ensemble but it takes a long time. However, this long time is not producing better results than Rxn-enum, seems pretty similar to me. I would use Rxn-enum because it goes really fast and produces that same accuracy as Diversity-enum. I am not sure if Diversity-enum produces valuable diversity within models. Unless authors can systematically show that extra context-specific metabolic networks that are being differentially captured by Diversity-enum but not by Rxn-enum are valuable. Authors can simply see which of the networks captured uniquely by Diversity-enum captured gene essentiality not explained by Rxn-enum.*

A-2.2 We show in the new evaluation using four different human cancer cell lines how `Diversity-enum` captures more diversity in terms of solutions that are enriched for alternative pathways. We also included additional figures showing differences between the methods (Figures 8 and 9 in the main manuscript) and

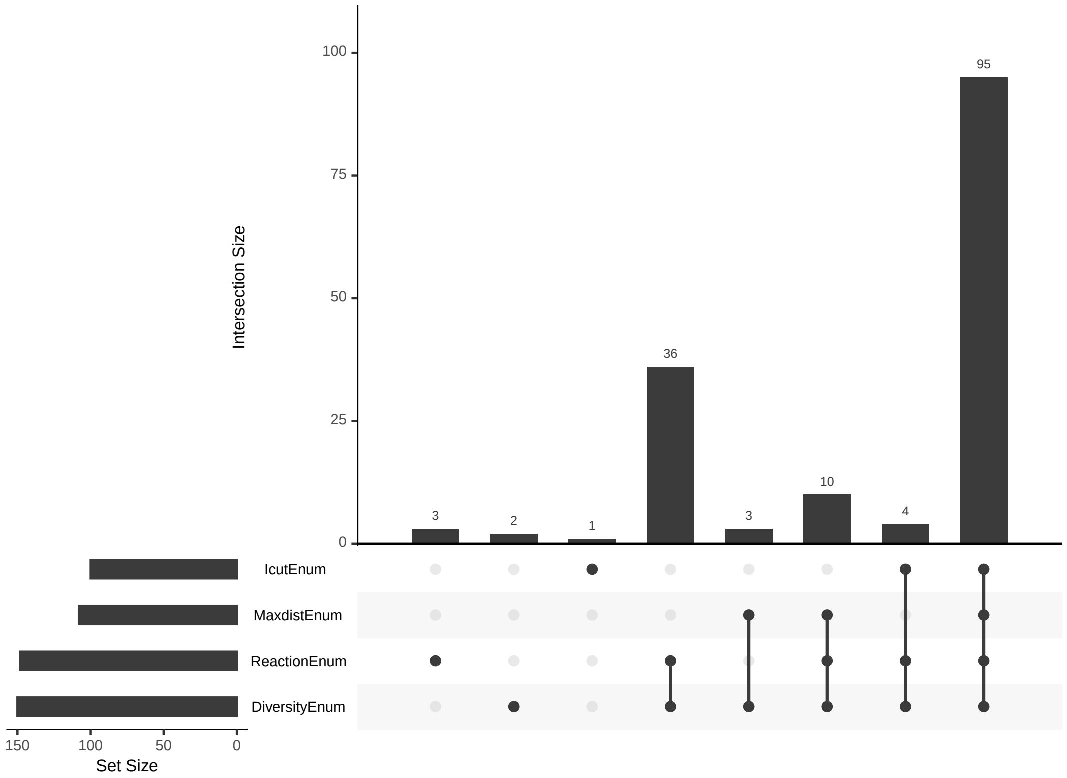

Figure 1: Intersection of the correctly predicted essential genes (true positives) using the Yeast 6 model and the UpSetR package. The first three vertical bars show the number of unique essential genes detected by the `Reaction-enum` method (3 genes), `Diversity-enum` method (2 genes), and `Icut-enum` method (1 gene). The remaining vertical bars show the intersections between two, three and the four methods.

also the proportion of times each pathway was detected as enriched by each method (including the variability between methods). These plots are included in the supplementary pdf (S1 Appendix). Also, in the case of essential genes, it can be seen also how `Diversity-enum` predicts genes that were not predicted by `Reaction-enum` (Fig. 1).

Q-2.3 *If Diversity-enum is better, could the authors just do a statistical test for difference in distributions of TPR from different methods? These are basically p-values from comparing TPR distributions between pairs of methods.*

A-2.3 In the paper we highlight how `Diversity-enum` generates the ensemble with the highest TPR (which is also true for different ds parameters). However, we think that the interesting result in this first analysis is the big difference between the two groups of techniques, not the differences between `Diversity-enum` and `Reaction-enum`. Indeed, we mention how both `Diversity-enum` and `Reaction-enum` behave on average (across thresholds) in a very similar way, as it can be seen in the TPR/FPR figure. We did not test for statistical significance because after looking at the trend of both methods we did not expect that these differences were significant. Although a simple t-test for comparing the mean TPR of both

techniques rejects the null hypothesis of no differences for an $\alpha = 0.05$ (p-value $= 0.0215$), we believe that some of the assumptions of the test might be violated. For example, each ensemble (point) is not an independent measurement, since all the models were built using the same gene expression data with different thresholds. On the other hand, the analysis would require some type of multivariate statistical test considering both the FPR and TPR at the same time and not only the TPR, but we have also a small sample (16 points per method) due to how costly it is in terms of computational time to do the evaluation. It is therefore that we have preferred to make only the observation that there is always a threshold in the tests we performed for which `Diversity-enum` returns an ensemble with a better TPR than any of the others ensembles produced by the other methods.

Response to Referee 3

Q-3.1 *The authors underwent substantial labor to improve their manuscript. The focus of the paper has been improved and shifted away from focusing on the new DEXOM formulation itself (now termed Diversity-enum) to presenting a suite of readily usable algorithms for enumerating alternative optimal metabolic network solutions. These include Diversity-enum as one of four methods for which no COBRA compatible toolbox extension was available so far. The precision in phrasing has been improved throughout the manuscript. More importantly the authors succeeded in adding another use-case, by adding an enrichment analysis of four human cancer cell lines. The authors showed that a set of differing context specific metabolic models with equal optimality can possess a diverse set of enriched metabolic pathways. This outcome is a valuable addition to the manuscript, but misses one aspect to be complete (see below). The authors show that equally probable context specific networks can possess a diverse set of enriched functions. In consequence pathway enrichment analysis needs to be treated with care. The authors do not show, however, how frequent any of these pathways occur, e.g. is there a single pathway that is enriched in most, if not in all, sets of networks? This way enriched pathways occurring only a few times, might be considered artifacts of the respective networks, yet pathways that are enriched in most networks hint towards metabolic function that translated into the context specific metabolic networks and thus would be of value. Here, it would also be of interest how the frequency of enriched pathways differs after applying the four different methods. Such an analysis should be easy to add, would improve the provided insights into enriched pathway analysis and complete the revision.*

A-3.1 We thank the reviewer for the feedback. We agree that showing the frequency of enriched pathways will provide an additional layer of information to the results. We thus decided to include those plots for each threshold and cell line evaluated. These figures were added at the end of the S1 Appendix pdf (the section called *"Variability in the detection of enriched pathways between the different methods"*). We also highlight how the different thresholds can have an important impact on these frequencies and we also discuss about the interpretation of the frequencies (which is difficult). We did also a similar analysis for the case of essential genes as well (section *"Distribution of predicted essential genes among methods"* in the S1 Appendix file).
